# Effect of pedometer-based walking interventions on long-term health outcomes: Prospective 4-year follow-up of two randomised controlled trials using routine primary care data

**DOI:** 10.1371/journal.pmed.1002836

**Published:** 2019-06-25

**Authors:** Tess Harris, Elizabeth S. Limb, Fay Hosking, Iain Carey, Steve DeWilde, Cheryl Furness, Charlotte Wahlich, Shaleen Ahmad, Sally Kerry, Peter Whincup, Christina Victor, Michael Ussher, Steve Iliffe, Ulf Ekelund, Julia Fox-Rushby, Judith Ibison, Derek G. Cook

**Affiliations:** 1 Population Health Research Institute, St George’s University of London, Tooting, London, United Kingdom; 2 Pragmatic Clinical Trials Unit, Queen Mary’s University of London, London, United Kingdom; 3 Gerontology and Health Services Research Unit, Brunel University, London, United Kingdom; 4 Institute for Social Marketing and Public Health, University of Stirling, Stirling, Scotland, United Kingdom; 5 Research Department of Primary Care and Population Health, University College London, London, United Kingdom; 6 Department of Sport Medicine, Norwegian School of Sport Sciences, Oslo, Norway; 7 Department of Population Science, King’s College London, London, United Kingdom; 8 Institute of Medical and Biomedical Education, St George’s University of London, Tooting, London, United Kingdom; Harvard Medical School, UNITED STATES

## Abstract

**Background:**

Data are lacking from physical activity (PA) trials with long-term follow-up of both objectively measured PA levels and robust health outcomes. Two primary care 12-week pedometer-based walking interventions in adults and older adults (PACE-UP and PACE-Lift) found sustained objectively measured PA increases at 3 and 4 years, respectively. We aimed to evaluate trial intervention effects on long-term health outcomes relevant to walking interventions, using routine primary care data.

**Methods and findings:**

Randomisation was from October 2012 to November 2013 for PACE-UP participants from seven general (family) practices and October 2011 to October 2012 for PACE-Lift participants from three practices. We downloaded primary care data, masked to intervention or control status, for 1,001 PACE-UP participants aged 45–75 years, 36% (361) male, and 296 PACE-Lift participants, aged 60–75 years, 46% (138) male, who gave written informed consent, for 4-year periods following randomisation. The following new events were counted for all participants, including those with preexisting diseases (apart from diabetes, for which existing cases were excluded): nonfatal cardiovascular, total cardiovascular (including fatal), incident diabetes, depression, fractures, and falls. Intervention effects on time to first event post-randomisation were modelled using Cox regression for all outcomes, except for falls, which used negative binomial regression to allow for multiple events, adjusting for age, sex, and study. Absolute risk reductions (ARRs) and numbers needed to treat (NNTs) were estimated. Data were downloaded for 1,297 (98%) of 1,321 trial participants. Event rates were low (<20 per group) for outcomes, apart from fractures and falls. Cox hazard ratios for time to first event post-randomisation for interventions versus controls were nonfatal cardiovascular 0.24 (95% confidence interval [CI] 0.07–0.77, *p* = 0.02), total cardiovascular 0.34 (95% CI 0.12–0.91, *p* = 0.03), diabetes 0.75 (95% CI 0.42–1.36, *p* = 0.34), depression 0.98 (95% CI 0.46–2.07, *p* = 0.96), and fractures 0.56 (95% CI 0.35–0.90, *p* = 0.02). Negative binomial incident rate ratio for falls was 1.07 (95% CI 0.78–1.46, *p* = 0.67). ARR and NNT for cardiovascular events were nonfatal 1.7% (95% CI 0.5%–2.1%), NNT = 59 (95% CI 48–194); total 1.6% (95% CI 0.2%–2.2%), NNT = 61 (95% CI 46–472); and for fractures 3.6% (95% CI 0.8%–5.4%), NNT = 28 (95% CI 19–125). Main limitations were that event rates were low and only events recorded in primary care records were counted; however, any underrecording would not have differed by intervention status and so should not have led to bias.

**Conclusions:**

Routine primary care data used to assess long-term trial outcomes demonstrated significantly fewer new cardiovascular events and fractures in intervention participants at 4 years. No statistically significant differences between intervention and control groups were demonstrated for other events. Short-term primary care pedometer-based walking interventions can produce long-term health benefits and should be more widely used to help address the public health inactivity challenge.

**Trial registrations:**

PACE-UP isrctn.com ISRCTN98538934; PACE-Lift isrctn.com ISRCTN42122561.

## Abstract 2

Tess Harris and colleagues reveal long-term benefits for pedometer-based walking interventions, with fewer CVD events and fractures 4 years after trial initiation.

## Author summary

**Why was this study done?:**

- Health benefits from physical activity interventions require sustained increases in physical activity levels, yet evidence of long-term objective increases in physical activity and effects on health outcomes is lacking.
- Routine data from primary care records can provide robust health outcome measures but have been little used in evaluating physical activity interventions.

**What did the researchers do and find?:**

- Two primary care 12-week pedometer-based walking interventions in 45- to 75-year-olds increased objectively measured moderate-to-vigorous physical activity levels by one-third between baseline and 3–4 years and were associated with significant decreases in both cardiovascular events (heart attacks, strokes, etc.) and fractures at 4 years.
- Approximately 60 people needed to receive the walking intervention to prevent one cardiovascular event and approximately 28 people to prevent one fracture.

**What do these findings mean?:**

- Short-term pedometer-based walking interventions can help adults and older adults to achieve not only sustained increases in physical activity but also important long-term health benefits.
- Routine primary care data provide an efficient way to provide long-term trial follow-up for assessing health outcomes.

## Introduction

Strong evidence exists that physical activity (PA) is protective for a wide range of health conditions [1–3], and inactivity is claimed to be the fourth leading risk factor for global mortality [2]. Meta-analyses of cohort studies have reported clear benefits of moderate-intensity PA for many chronic diseases, including diabetes [4], ischaemic heart disease [4], stroke [4], fractures [5], and depression [6]. However, all these estimates are based on evidence from observational cohort studies, in which individual baseline differences in PA levels, assessed by questionnaires, were linked to subsequent disease outcomes. PA questionnaires are known to be inaccurate and subject to recall bias [7], and there is the possibility of regression dilution bias in such studies, which would lead to underestimating benefits. This raises the question of whether changes in PA that occur after PA interventions will have similar or possibly larger effects. An additional advantage of trial data is that changes in PA have usually been objectively measured.

Many PA interventions, including pedometer-based walking interventions, have shown short-term increases in PA levels [8–10], but to achieve the long-term health benefits demonstrated above in cohort studies, increases in PA need to be sustained, and long-term trial data with objectively measured PA outcomes and robust health outcomes are limited, with calls for more such trials [10–12]. The Finnish Diabetes Prevention Study observed significant PA increases and showed impressive 58% reductions in type 2 diabetes, but it combined dietary and PA interventions; therefore, it is difficult to estimate independent PA effects [13]. In contrast, a recent large primary care trial failed to reduce type 2 diabetes incidence, despite increasing PA levels [14]. Trials examining PA effects on cardiovascular outcomes have shown mixed results: some showing strong protective effects on both heart attacks [15,16] and strokes [16] but others failing to reduce cardiovascular events [17]. Primary care trials that have successfully increased PA levels have shown both increased [18] and reduced [19] self-reported falls, but a systematic review of exercise interventions in older adults demonstrated a significant reduction in fall-related fractures [20]. A meta-analysis of PA interventions demonstrated a reduction in depressive symptoms [21]. However, only one of the trials above used routinely recorded primary care or hospital data to capture events [15]; for other trials, participants were asked about health events, and these were validated by checking patient records, leading to important potential reporting bias. The benefit of using routine electronic records for extended follow-up of trials has been established in a different context by the West of Scotland Coronary Prevention Study, which showed that routine records gave very similar results to rigorously collected clinical trial data for cardiovascular events and deaths [22]. Using routine records in trials has the additional merit that if participants have given permission to access their health records, these may be available even when participants are lost to follow-up.

We conducted two pedometer-based walking trials with adults and older adults (PACE-UP, PACE-Lift), which increased accelerometer-measured step count and moderate-to-vigorous PA (MVPA) levels in bouts at 12 months [23,24] and with sustained PA increases at 3–4 years [25]. Across both trials at 3–4 years, all intervention groups were doing approximately an extra 30 minutes per week of MVPA compared with baseline, up about a third on their baseline levels [25]. Both trials recruited through primary care, and participant consent to link trial data with primary care record data was sought. The aim of the present paper was to evaluate the intervention effects from PACE-UP and PACE-Lift on longer-term health outcomes relevant to the walking interventions, using routinely collected primary care data.

## Methods

### Study design and participants

Two primary care randomised controlled trials (RCTs) of effective 12-week pedometer-based walking interventions are included, both providing long-term health outcome data from routine primary care records: PACE-UP, which recruited 45- to 75-year-olds from seven London (United Kingdom) practices from October 2012 to November 2013, and PACE-Lift, which recruited 60- to 75-year-olds from three Berkshire and Oxfordshire (UK) practices from October 2011 to October 2012. The trials were similar in their primary care recruitment and in the 12-week pedometer-based walking interventions, incorporating behaviour-change techniques [26, 27]. Participants were very similar in terms of their baseline characteristics, apart from deprivation level (see Table 1), but randomisation ensured even distribution of deprivation levels across intervention and control groups [23,24], and deprivation was not an effect modifier [23]. At long-term follow-up, 3-year findings for both PACE-UP intervention groups (postal and nurse-supported) and 4-year findings from the PACE-Lift (nurse-supported) intervention group all showed very similar effects on PA levels [25]. Given the similarities of these trials and their sustained PA effects, we therefore present a combined analysis of all three intervention groups at 4 years on primary care outcomes. All analyses adjust for study as a covariate.

**Table 1:** Baseline characteristics of the PACE-UP and PACE-Lift cohorts.

	PACE-UP study (*N* = 1,001)	PACE-Lift study (*N* = 297)^a^
	*n* (%)	*n* (%)
**Age at randomisation**		
45–59 years	520 (52%)	0 (0%)
60–75 years	481 (48%)	297 (100%)
**Gender**: male	361 (36%)	138 (46%)
**Marital status**: married	645 (66%)	240 (81%)
**National quintiles of Index of Multiple Deprivation rank**		
1–3 (most deprived)	553 (57%)	29 (10%)
4	212 (22%)	51 (17%)
5 (least deprived)	201 (21%)	217 (73%)
**Ethnicity**		
White	776 (80%)	289 (99%)
Asian/Asian British	68 (7%)	2 (1%)
Black/African/Caribbean/Black British	96 (10%)	1 (0%)
Other	24 (2%)	1 (0%)
**Current smoker**	80 (8%)	16 (6%)
**General health**^b^: Very good or good	802 (82%)	259 (89%)
**Self-reported pain**^b^	674 (69%)	201 (69%)
**Limiting long-standing illness**	216 (22%)	72 (26%)
**Townsend disability score**^b^		
None (0)	586 (59%)	205 (70%)
Slight or some disability (1–6)	371 (38%)	83 (28%)
Appreciable or severe disability (7–18)	29 (3%)	6 (2%)
**HADS depression score**^b^: borderline or high	108 (11%)	
**Geriatric depression score**^b^: high		19 (7%)
**Overweight/obese**: BMI ≥ 25kg/m^2^	666 (67%)	200 (67%)
**Preexisting disease on GP records**		
Cardiovascular disease	64 (6%)	31 (10%)
Diabetes	70 (7%)	18 (6%)
Depression	99 (10%)	34 (11%)
**Accelerometry data**		
**Average adjusted baseline step count per day**		
Mean (sd)	7,492 (2,675)	7,331 (2,829)
Median (IQR)	7,344 (5,567–9,106)	7,043 (5,302–9,124)
**Total weekly minutes of MVPA in ≥10-minute bouts**		
Mean (sd)	94 (102)	92 (109)
Median (IQR)	65 (21 to 133)	53 (3 to 140)

^a^One PACE-Lift participant in the control group died before 12 months and is included in this table. The participant is not included in the analysis of primary care record data but is included in the fatal + nonfatal cardiovascular events analysis.

^b^Full references for general health, self-reported pain, Townsend disability score, HADS score, and geriatric depression score are given in the trial protocols [26,27].

Abbreviations: BMI, body mass index; GP, general practitioner; HADS, hospital anxiety and depression scale; IQR, interquartile range; MVPA, moderate to severe physical activity.

#### PACE-UP trial

Trial methods are published [27], as are 3- and 12-month [23] and 3-year [25] findings, cost-effectiveness analyses [28], and funder’s report [29]. All research participants gave written informed consent, and permission was sought for researchers to access data from their primary care records. Ethical approval was granted by London Hampstead Research Ethics Committee (REC) (UK) (12L/LO/0219), including substantial amendments for extended follow-up work. The trial had a control arm (usual care) and two intervention arms: postal, who received the 12-week PACE-UP walking programme (pedometer, handbook, and PA diary) by post, and nurse-supported, who received the same materials at the first of three practice nurse PA consultations. Baseline findings for participants included in primary care outcomes analyses are summarised in Table 1, and the postal and nurse interventions are summarised in Table 2. The handbook and diary are available at www.paceup.sgul.ac.uk/materials. The protocol, approved by ethics before extended follow-up commenced, included details of long-term follow-up, primary care data download procedures, and primary care data outcomes and is included (S1 Text), as is a CONSORT checklist for primary care data follow-up (S2 Text).

**Table 2:** Components of interventions for PACE-UP and PACE-Lift trials^a^.

Component	PACE-UP		PACE-Lift
	Postal	Nurse	Nurse
**Pedometer** Yamax Digi-Walker (Tokyo, Japan) SW-200. Provides step count, requires daily manual recording and resetting	Posted with instructions for use^b^	Given with instructions by nurse at first appointment	Given with instructions by nurse at first appointment
**Dedicated practice nurse PA consultations** (including behaviour-change techniques)	Not applicable	3 consultations	4 consultations,
		Week 1 “First Steps” (approx. 30 mins)	Week 1 “First Steps” (approx. 45 mins)
		Week 5 “Continuing the Changes” (approx. 20 mins)	Week 3 “Continuing the Changes” (approx. 30 mins)
		Week 9 “Building Lasting Habits” (approx. 20 mins)	Week 7 “Keeping up the Changes” (approx. 30 mins)
			Week 11 “Building Lasting Habits” (approx. 30 mins)
**Accelerometer feedback as part of intervention**	Not applicable	Not applicable	Actigraph GT3X+ (accelerometer) worn for 1 week prior to each nurse appointment. Nurse downloaded accelerometer data during consultation and provided immediate feedback on time spent in sedentary, light, moderate, and vigorous PA levels in relation to activities recorded in PA diary.
**Handbook** (including behaviour-change techniques)	Posted^b^	Given by nurse at first appointment	Given by nurse at first appointment
**Target-setting: step count goals and PA goals and use of walking planner**	Blinded pedometer (Yamax DigiWalker CW200) worn for 7 days at baseline to calculate average daily baseline steps, used to set step count targets. Use of 12-week walking planner. Advised to add 1,500 steps/day and then 3,000 steps/day to average baseline steps in graded manner over 12 weeks.	Blinded pedometer (Yamax DigiWalker CW200) worn for 7 days at baseline to calculate average daily baseline steps, used to set step count targets. Use of 12-week walking planner. Advised to add 1,500 steps/day and then 3,000 steps/day to average baseline steps in a graded manner over 12 weeks.	Nurses discussed appropriate step count and PA goals with participants based on baseline step count and weekly time in MVPA from accelerometry and any health issues. Participants encouraged to set both step count and time in MVPA goals, encouraged to start low and go slow. Walking planner to help them plan when and where and with whom they planned to walk. Goals reviewed and reset at each consultation.
	“3,000-steps-in-30-minutes” message for PA intensity.	Targets could be adapted in discussion with nurse.	
		“3,000-steps-in-30-minutes” message for PA intensity.	
**12-week PA and step count diary** (including behaviour-change techniques)	Posted^b^ and encouraged to return completed diary to researchers after 12-week intervention.	Given by nurse at first appointment, reviewed by nurse at other appointments and encouraged to return completed diary to researchers after 12-week intervention.	Given by nurse at first appointment and reviewed at each nurse appointment.

^a^This table has been adapted from tables in the published trial protocols [26,27]. These are open-access articles published under licence to BioMed Central and distributed under the terms of the Creative Commons Attribution License, which permits unrestricted use, distribution, and reproduction, provided the work is properly cited (http://creativecommones.org/licenses/by/2.0).

^b^Researcher telephoned 1 week later to check whether programme had arrived.

Abbreviations: approx., approximately; MVPA, moderate-to-vigorous PA; PA, physical activity.

#### PACE-Lift trial

Trial methods are published [26], as are 3- and 12-month [24] and 4-year [25] findings. Written informed consent was gained from all participants, and permission was sought for researchers to access data from participants’ primary care records. Initial ethical approval was granted by Oxfordshire REC C (UK) (11/H0606/2) up to 12-month follow-up; the study was then closed and new ethical approval from this REC was granted for 4-year follow-up (15/SC0352), which required us to reconsent participants at 4 year to access further data from their primary care records, as trial follow-up was being restarted. The trial had a control arm (usual care) and an intervention arm whose participants received the 12-week PACE-Lift walking programme (pedometer, handbook, PA diary, and feedback on their accelerometry measures) at the first of four practice nurse PA consultations. Baseline findings for participants included in primary care outcome analyses are summarised in Table 1, and the intervention is summarised in Table 2. The protocol, approved by ethics before extended follow-up commenced, included details of long-term follow-up, primary care data download, and primary care data outcomes and is included (S3 Text), as is a CONSORT checklist for primary care data follow-up (S4 Text).

### Sample size

Power calculations for each of the trials have been described previously in the PACE-UP [27] and PACE-Lift [26] trial protocols and were related to the primary outcome of change in objectively measured PA levels at prespecified time points. The sample size was determined by the number of trial participants providing consent for primary care data download, and the confidence intervals (CIs) around our estimates give a clear indication of the level of precision of our findings.

### Outcome measures

A priori we wanted to evaluate intervention effects on long-term health outcomes relevant to walking interventions assessed from primary care records 4 years post-baseline. These outcome measures were not described in the original published trial protocols [26, 27], as they were not planned at the time of study design, but they were prespecified in the protocols approved by ethics (S1 and S3 Text), ahead of long-term data collection, and were designed to be measurable retrospectively from primary care data. We defined these health outcomes as nonfatal cardiovascular events (myocardial infarction, coronary artery bypass graft, angioplasty, transient ischaemic attack, and stroke), total cardiovascular events (cardiovascular deaths plus nonfatal cardiovascular events), new onset diabetes, new onset depression episode, injurious falls (those recorded in primary care records), and fractures. Cardiovascular disease and depression outcomes were estimated both for those with and without prior cardiovascular disease or depression, respectively. We also examined the effect of the interventions on number of primary care consultations (excluding the 3-month intervention period, as consultations were part of the nurse intervention arms for both trials).

### Procedures

**Fig 1:**
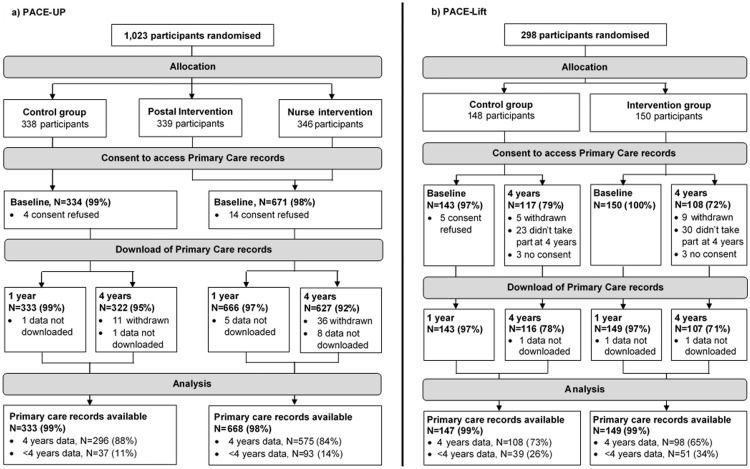
(a) PACE-UP and (b) PACE-Lift CONSORT diagrams for primary care records analyses. Complete data for 4 years were only available on those participants who were registered at the same primary care practice at baseline and 4 years. 7 deaths in PACE-UP participants (2 Control. 5 Intervention) and 4 deaths in PACE-Lift participants (2 Control. 2 Intervention) were recorded on registered patients. Data for those participants who moved away or had died before 4 years are censored when they left the practice or at their date of death.

We downloaded primary care data for trial participants who gave written consent, minus those who subsequently withdrew from the trials, for the 4-year periods following randomisation from the seven PACE-UP and three PACE-Lift practices. Data were downloaded at 12 months (at end of initial trial follow-up) and at 4 years (after extended follow-up) at all 10 practices. If participants had both sets of data, the 12-month data were not needed, as they were duplicated in the 4-year data. For those without any data at 4 years, 12-month data were used, if available. See CONSORT diagram (Fig 1) for details of numbers of participants at each time point with primary care data for both trials. Data on participants were censored if a patient left the practice or if they died whilst still registered at the practice. Searches were set up to download the following information from primary care records: Read codes for diseases (including those arising from hospital admissions) and consultation data (see S1 Fig for details of exactly how events were counted). For cardiovascular events and depression, we separated events occurring in participants with and without preexisting disease, so Read codes for these diseases that occurred prior to the individual randomisation date were taken as evidence of preexisting disease. Prior Read codes for diabetes were used to exclude those with preexisting diabetes at date of randomisation from subsequent diabetes analyses only, so that we could estimate numbers of new type 2 diabetes diagnoses. Data were processed blind to trial group, by a researcher without access to trial data apart from randomisation dates, according to an agreed protocol. Two medically qualified researchers, also blind to intervention group, separately checked that the events counted were appropriate. Details on deaths (including date and cause) were collected systematically for both trials from general practices prior to recontacting participants for long-term follow-up (at 3 years for PACE-UP and 4 years for PACE-Lift) and were therefore available for all participants for this period of follow-up (regardless of whether they had given permission for their primary care records to be downloaded), apart from any patients who had deregistered with their practice by moving away. Known deaths from cardiovascular causes were counted as outcome events and included in total cardiovascular events; other deaths were treated as censored data. Once all events had been verified, the primary care data and trial data were linked.

### Statistical analysis

Analyses were carried out in STATA version 15.0 (StataCorp). We combined data from both trials and modelled the effect of the interventions on cardiovascular, diabetes, fracture, and depression outcomes using Cox regression models to estimate Cox hazard ratios (and 95% CIs) for time to first event post-randomisation, adjusting for age (as a continuous variable), sex, and study. For cardiovascular disease and depression, we repeated analyses excluding those with a prior cardiovascular disease or depression diagnosis. For all these outcomes, we also calculated Kaplan-Meier survival curves. For falls and consultations, in which we wanted to allow for multiple events to be counted, we used negative binomial regression, which models the counts as a Poisson process but allows for overdispersion. These models were used to estimate incident rate ratios (and 95% CIs), also adjusting for age, sex, and study. We estimated absolute risk reductions (ARRs) and used the approach recommended by Altman to calculate the number needed to treat (NNT) for each estimate and its confidence limits [30, 31].

### Patient and public involvement

Pilot work with older primary care patients from three general practices was carried out prefunding, with focus groups discussing ideas for a pedometer-based PA intervention. They provided input into study design—for example, encouraging postal recruitment and recruitment of couples as well as individuals. Both trials had a patient advisor as a Trial Steering Committee member; they were involved in discussions about study conduct and advised on patient materials, dissemination of results to participants, and safety reporting. All trial participants were provided with individual feedback after 12-month follow-up. Trial results were disseminated at the following times: after baseline assessments, after analysis of the main 12-month results, and after 3-year (PACE-UP) or 4-year (PACE-Lift) follow-up. A trial website summarising trial results and publications was created for PACE-UP (http://www.paceup.sgul.ac.uk) and circulated to participants. Intervention burden was assessed by nurse group participants as part of trial process evaluations [32] and by samples of all the intervention groups as part of the qualitative evaluations [33, 34].

## Results

Overall, 98% (1,297/1,321) of initial trial participants gave written consent for primary care data linkage and had their data downloaded—1,001/1,023 (98%) from PACE-UP and 296/298 (99%) from PACE-Lift at baseline—and 223/225 (99%) of PACE-Lift participants recontacted at 4-year follow-up. Primary care data available at different time points are shown in the two flow diagrams (Fig 1a and 1b). Overall, 82% (1,077/1,321) of participants had 4 years of complete data: 85% (871/1,023) of PACE-UP and 69% (206/298) of PACE-Lift participants.

**Fig 2:**
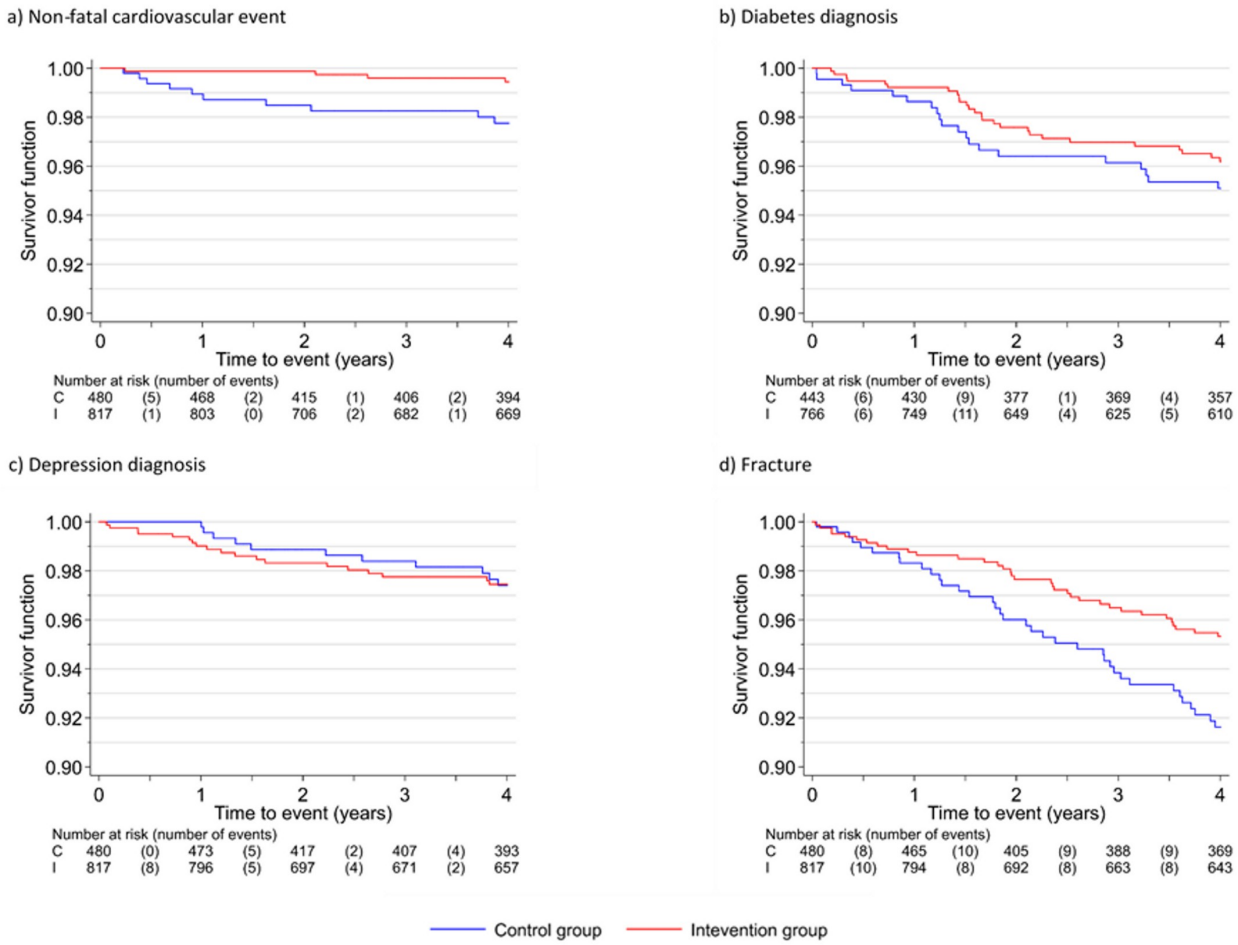
Kaplan-Meier curves for time to first event post-randomisation recorded in primary care records.

Table 3 presents the time to first event for each outcome; the model coefficients are given in S1 Table. For nonfatal cardiovascular events, in both trials the proportion of events was lower in the intervention than in the control group, both for those without a prior cardiovascular diagnosis and for all participants. The hazard ratios for all participants were 0.24 (95% CI 0.07–0.77, *p* = 0.02) (demonstrated in a Kaplan-Meier plot in Fig 2) and for those without a prior diagnosis 0.27 (95% CI 0.08–0.88, *p* = 0.03). When fatal cardiovascular events were included, results were similar: hazard ratios of 0.34 (95% CI 0.12–0.91, *p* = 0.03) for all participants and 0.31 (95% CI 0.11–0.93, *p* = 0.04) for those without a prior diagnosis. In terms of new diabetes diagnoses, there was no statistically significant intervention effect, with a hazard ratio of 0.75 (95% CI 0.42–1.36, *p* = 0.34). Similarly, for new depression diagnoses, there was no overall effect of the intervention, hazard ratio 0.98 (95% CI 0.46–2.07, *p* = 0.96) in all participants and 0.92 (95% CI 0.41–2.03, *p* = 0.83) in those without a prior diagnosis. For fractures, in PACE-UP the proportion of patients with a fracture during follow-up was lower in the intervention group (26/668, 3.9%) compared with the control group (28/333, 8.4%); for PACE-Lift both groups had similar proportions of fractures, 5.4% (8/149) in the intervention group and 5.4% (8/147) in the control group. The overall Cox regression hazard ratio across both trials was significantly reduced (0.56, 95% CI 0.35–0.90, *p* = 0.02). Fig 2 also shows these findings in a Kaplan-Meier time-to-first-event diagram.

**Table 3:** PACE-UP and PACE-Lift studies. Hazard ratios for first events after randomisation in primary care records.

	PACE-UP			PACE-Lift^a^			PACE-UP and PACE-Lift			HRs^b^	
Event Recorded in Primary Care Record	*N*	Control (*n* = 333)	Intervention (*n* = 668)	*N*	Control (*n* = 147)	Intervention (*n* = 149)	*N*	Control (*n* = 480)	Intervention (*n* = 817)	HR (95% CI)	*p*-Value
**Nonfatal cardiovascular events**											
All participants	1,001	7 (2.1%)	3 (0.4%)	296	3 (2.0%)	1 (0.7%)	1,297	10 (2.1%)	4 (0.5%)	0.24 (0.07–0.77)	0.02
No previous cardiac diagnosis	937	6 (1.9%)	3 (0.5%)	265	3 (2.3%)	1 (0.8%)	1,202	9 (2.0%)	4 (0.5%)	0.27 (0.08–0.88)	0.03
**Nonfatal and fatal cardiovascular events**											
All participants	1,001	7 (2.1%)	4 (0.6%)	297	4 (2.7%)	2 (1.3%)	1,298	11 (2.3%)	6 (0.7%)	0.34 (0.12–0.91)	0.03
No previous cardiac diagnosis	937	6 (1.9%)	3 (0.5%)	266	4 (3.0%)	2 (1.5%)	1,203	10 (2.3%)	5 (0.7%)	0.31 (0.11–0.93)	0.04
**Diabetes diagnosis**											
No previous diabetes diagnosis	931	16 (5.2%)	21 (3.4%)	278	4 (2.9%)	5 (3.5%)	1,209	20 (4.5%)	26 (3.4%)	0.75 (0.42–1.36)	0.34
**Depression diagnosis**											
All participants	1,001	11 (3.3%)	15 (2.2%)	296	0 (0.0%)	4 (2.7%)	1,297	11 (2.3%)	19 (2.3%)	0.98 (0.46–2.07)	0.96
No previous depression diagnosis	902	10 (3.3%)	13 (2.2%)	262	0 (0.0%)	3 (2.3%)	1,164	10 (2.3%)	16 (2.2%)	0.92 (0.41–2.03)	0.83
**Fractures**											
All participants	1,001	28 (8.4%)	26 (3.9%)	296	8 (5.4%)	8 (5.4%)	1,297	36 (7.5%)	34 (4.2%)	0.56 (0.35–0.90)	0.02

^a^One PACE-Lift participant in the intervention group died before 12 months, and primary care records are not available. This participant has only been included in the analyses of nonfatal + fatal cardiovascular events, so the denominators for these analyses are 150 for the PACE-Lift intervention group and 818 for the combined intervention group.

^b^Estimates from the Cox regression models are adjusted for age, sex, and study.

Abbreviation: HR, hazard ratio.

Table 4 shows the findings for medically reported falls and consultations; S1 Table shows the coefficients. Overall, approximately 14% (115/818) of intervention and 13% (63/480) of control participants had one or more falls during follow-up; the overall incident rate ratio for falls was 1.07 (95% CI 0.78–1.46, *p* = 0.67). The mean number of consultations per year over 4-year follow-up was similar in intervention and control groups for both trials (approximately 6, with a standard deviation of 5), and the incident rate ratio was 1.01 (95% CI 0.93–1.10, *p* = 0.82), showing no intervention effect on number of consultations.

**Table 4:** PACE-UP and PACE-Lift studies. Incident rate ratios for falls and consultations after randomisation from primary care records.

	PACE-UP		PACE-Lift		PACE-UP and PACE-Lift			
	Control	Intervention	Control	Intervention	Control	Intervention	Incident Rate Ratio^a^	
	(*n* = 333)	(*n* = 668)	(*n* = 147)	(*n* = 149)	(*n* = 480)	(*n* = 818)	IRR (95% CI)	*p*-Value
**Number of falls**								
	*n* (%)	*n* (%)	*n* (%)	*n* (%)	*n* (%)	*n* (%)		
0	285 (86%)	574 (86%)	132 (90%)	128 (86%)	417 (87%)	702 (86%)		
1	39 (12%)	80 (12%)	10 (7%)	15 (10%)	49 (10%)	95 (12%)		
2	8 (2%)	11 (2%)	2 (1%)	3 (2%)	10 (2%)	14 (2%)		
3+	1 (0.3%)	3 (0.3%)	3 (2%)	3 (2%)	4 (1%)	6 (1%)		
**Falls and consultations: Rate per year of follow-up**								
	Mean (sd)	Mean (sd)	Mean (sd)	Mean (sd)	Mean (sd)	Mean (sd)		
**Falls**	0.04 (0.12)	0.05 (0.14)	0.04 (0.14)	0.05 (0.15)	0.04 (0.13)	0.05 (0.14)	1.07 (0.78–1.46)	0.67
**Consultations**^b^	5.9 (4.8)	6.1 (5.1)	6.1 (4.6)	6.2 (5.9)	5.9 (4.8)	6.1 (5.3)	1.01 (0.93–1.10)	0.82

^a^Negative binomial models were used to estimate incident rate ratios. These model the count as Poisson with extra variation; the dispersion parameter is the expected mean. All models adjust for age, sex, and study.

^b^Consultations in the first 90 days after randomisation are not included, as intervention group participants in both studies had practice nurse consultations during this time as part of the intervention.

Table 5 shows the ARRs and NNTs (95% CIs) for each event, combined across both trials. The results for cardiovascular events and fractures (for which our interventions were associated with a protective effect) for all participants were as follows: nonfatal cardiovascular events ARR 1.7% (0.5%–2.1%), NNT 59 (48–194); total cardiovascular events ARR 1.6% (0.2%–2.2%), NNT 61 (46–472); fractures ARR 3.6% (0.8%–5.4%), NNT 28 (19–125).

**Table 5:** PACE-UP and PACE-Lift studies combined: ARRs and NNTs for primary care recorded events.

Event recorded in primary care record	*N*	ARR^a^ (95% CI)	NNT^b^ (95% CI)
**Nonfatal cardiovascular events**			
All participants	1,297	1.7 (0.5–2.1)	59 (48–194)
No previous cardiac diagnosis	1,202	1.6 (0.3–2.0)	62 (50–386)
**Nonfatal and fatal cardiovascular events**			
All participants	1,298	1.6 (0.2–2.2)	61 (46–472)
No previous cardiac diagnosis	1,203	1.7 (0.2–2.2)	60 (46–562)
**Diabetes diagnosis**			
No previous diabetes diagnosis	1,209	1.2 (−1.7 to 2.8)	84 (NNTH 59 to ∞ to NNTB 35)
**Depression diagnosis**			
All participants	1,297	0.1 (−2.7 to 1.4)	1,873 (NNTH 37 to ∞ to NNTB 72)
No previous depression diagnosis	1,164	0.2 (−2.6 to 1.5)	463 (NNTH 39 to ∞ to NNTB 66)
**Fractures**			
All participants	1,297	3.6 (0.8–5.4)	28 (19–125)

^a^ARR is calculated as 1/NNT.

^b^NNT/NNTB is the number needed to treat to show benefit from the intervention (i.e., prevent one event) at 4 years. Where the 95% CI for ARR is consistent with an increase in risk from the intervention, the NNTH is also shown [31]. NNT is calculated using the formula 1/{SurvC**HR–SurvC} where SurvC is the Kaplan-Meier survival probability in the control group at 4 years and HR is the hazard ratio [30].

Abbreviations: ARR, absolute risk reduction; NNT, number needed to treat; NNTH, number needed to harm.

## Discussion

### Statement of principal findings

Ninety-eight percent of trial participants gave written consent for their primary care records to be used and had data successfully downloaded. Our results indicate that 12-week pedometer-based walking interventions delivered through primary care, which led to long-term PA increases of approximately 30 minutes per week of MVPA in the intervention groups, were associated with significant decreases in both new cardiovascular events and fractures at 4 years. NNTs to avoid an event were approximately 60 for a cardiovascular event and 28 for a fracture. There was no intervention effect on number of consultations, suggesting that changes in consulting affecting recording of events could not explain these results. Our findings are important because they demonstrate long-term clinical benefits that apply to all those randomised, not only to those with trial follow-up data; clinical benefit also argues against explaining the PA differences at different time points as only being short-term changes during weeks when participants knew their PA levels were being measured. Our study thus demonstrates the advantage of using routine data to evaluate long-term health outcomes in trials and also in subsequent implementation studies.

### Strengths and weaknesses of the study

Important strengths were that both trials recruited from primary care and that we sought participant consent to use their primary care data, thus allowing us to examine long-term health outcomes using routinely collected data. Having two trials with similar recruitment methods and interventions, which we had previously combined for long-term follow-up [25], meant that we increased the power of our analyses. A key strength of UK routinely recorded primary care data, which may not apply to other healthcare systems, is that it fully captures secondary care diagnoses from accident and emergency, outpatients, and hospital admissions (National Health Service and private), in addition to primary care consultations and diagnoses, thus reducing potential for bias in outcome assessment. The inevitable losses that occur when collecting long-term trial follow-up data can be reduced by using routinely collected data for outcome assessment. We followed up 67% (681/1,023) of participants at 3 years in PACE-UP [25] and 76% (225/298) at 4 years for PACE-Lift with objective PA primary outcome data, but for these analyses using routinely collected primary care data at 4 years, we had 98% (1,297/1,321) contributing to analyses and 82% (1,077/1,321) providing complete data over the whole 4 years, despite needing to reconsent PACE-Lift participants at 4-year follow-up. Others have shown how higher levels of electronic follow-up (93%) compared with fieldwork follow-up (83%) at 2 years retained more of those in the most deprived groups [35], demonstrating reduced potential for selection bias.

An important limitation is that we did not have complete 4 years of follow-up data for all participants; however, differences were already evident in cardiovascular events and fractures by 12-month follow-up, when we had over 97% complete primary care data across all groups. We were also constrained by the data routinely recorded in primary care records, which do not reflect all cases. For cardiovascular events and fractures, underrecording is unlikely to have been a problem, as major diagnoses like myocardial infarction, stroke, coronary artery bypass graft, fractures, etc., are well recorded in primary care records following hospital notification of events. For falls and new depression episodes, cases occurring in the community but not reported to primary care will be missed, and for diabetes, new cases are only diagnosed when blood tests are done. However, although cases will be underrecorded in primary care records for these conditions, there is no reason for this to differ by intervention status, particularly as the intervention did not affect consultation rate, so this should not have led to bias. A further limitation is the uncertainty of our estimates. Given the low event rate in this sample and the relatively short follow-up period for events, the CIs are wide, indicating uncertainty regarding the exact magnitudes of effect.

### Comparisons with previous studies

Details of all the intervention studies that are referred to in this section, including more information on the specific interventions and their effects on PA levels, are summarised in S2 Table.

#### Cardiovascular events

The reductions we demonstrated in nonfatal (0.24 [95% CI 0.07–0.77]) and total (0.34 [95% CI 0.12–0.91]) cardiovascular events are consistent with the effect in older primary care patients 2 years after a 9-month PA programme that significantly increased self-reported PA and significantly decreased blood pressure and lipids (reduction in cardiovascular events risk ratio 0.15 [95% CI 0.04–0.51]) [15]. Similarly, others showed significant reductions in both heart attacks (relative risk 0.51) and strokes (relative risk 0.52) alongside self-reported PA increases, 6 months post-intervention in community-based hypertension patients [16]. However, Newman et al found no cardiovascular event reduction in the PA group at 2.6-year follow-up in older adults with functional limitations (hazard ratio 1.10 [95% CI 0.85–1.42]) [17]. Possible reasons were that the PA intervention group had more opportunity to report events; cardiovascular disease levels were high, possibly precipitating events or reducing potential benefits; and there was a possible suboptimal activity dose, as their moderate PA cutoff was >760 counts/minute [17], much lower than ours (≥1,952 counts/minute) [26, 27].

Comparisons with risk estimates from cohort studies are more difficult. A key issue is that all cohort studies in systematic reviews are based on questionnaire PA measures, with their known inaccuracies and recall bias [7]; the variety of different questionnaires also makes it difficult to standardise how PA is quantified. Cohort studies’ effect estimates usually compare inactive participants with those achieving much higher PA levels. Thus, a recent good-quality systematic review provides relative risks for an 11.25 metabolic equivalent (MET) hour/week increase in PA levels compared with being inactive of 0.69 (95% CI 0.67–0.71) [36], corresponding to international recommendations of 150 minutes of MVPA weekly in ≥10-minute bouts [37]. Our intervention increased PA by about 30 minutes of MVPA in bouts weekly long-term, one-fifth of 150 minutes, so after scaling down the relative risks from that paper [36] (S5 Text), their cardiovascular incidence effect estimate for the same level of PA increase becomes 0.98 (95% CI 0.97–0.99), suggesting a much more modest effect on cardiovascular events than from our intervention study.

#### Type 2 diabetes

We showed a nonsignificant reduction in type 2 diabetes cases (0.75 [95% CI 0.42–1.36]), consistent with a pedometer intervention in UK primary care prediabetes patients, which increased PA by 498 steps/day (162–834) across the 3-year follow-up and achieved a hazard ratio for type 2 diabetes cases of 0.74 (95% CI 0.48–1.14) [14]. The significant 58% reduction in diabetes incidence seen in the Finnish Diabetes Prevention trial (with a combined PA/dietary intervention) occurred with not only an increase in PA levels (86% of the intervention group achieving >4 hour/week of exercise compared with 71% of controls, *p* = 0.001) but also dramatic weight loss (4.2 ± sd 5.1 kg in the intervention group versus 0.8 ± sd 3.7 kg in controls) [13].

From cohort studies, the scaled-down estimate of effect for type 2 diabetes incidence (as done for cardiovascular disease, above) to compare appropriately with the PA increase achieved in our trials was 0.93 (95% CI 0.92–0.93) (S5 Text), consistent with our estimate.

#### Fractures

The reduction in fractures observed in the intervention groups compared with controls 0.56 (95% CI 0.35–0.90) is consistent with findings from a systematic review of exercise interventions in older adults that gave a pooled risk ratio of 0.60 (95% CI 0.45–0.85) [20] for fall-related fractures. However, only one of the 15 included studies was a pure walking intervention, and several studies focused on strength and balance training and did not include any walking component [20].

A meta-analysis of 13 cohort studies reported a relative risk for fractures of 0.62 (95% CI 0.56–0.69) in women [5] and 0.55 (95% CI 0.44–0.69) in men [5]. As for cardiovascular disease, these benefits are based on varying PA levels at baseline, but generally considerably greater than 30 minutes of MVPA from walking weekly, and thus need scaling back. Given the heterogeneity of exposure difference in the meta-analysis, it is difficult to provide such a scaled-back estimate.

#### Falls

We did not observe any reduction in medically reported falls (overall negative binomial regression incident rate ratio 1.07 [95% CI 0.78–1.46]). However, only primary care–recorded falls were captured. Although others have seen falls reduced by exercise interventions 0.86 (95% CI 0.77–0.94) [20] and 0.77 (95% CI 0.71–0.83) [38], most interventions included balance, resistance, and strength training and not simply walking. Walking is something of a paradox for risk of falling, as it can increase falls by increasing exposure. Those interventions that have reduced falls successfully in older adults have usually additionally included balance components [19, 20, 38], whereas pure walking interventions have sometimes increased falls [18], though not always [39].

A cohort study examining the association between regular walking and falls amongst community-dwelling older adults found that it did not increase falls for those at low risk (hazard ratio 0.88 [95% CI 0.48–1.62]), but it significantly increased risk in those with two or more risk factors for falling (hazard ratio 1.89 [95% CI 1.04–3.43]) [40], suggesting that the relationship between walking and falls is complex.

#### Depression

We found no intervention effect on new depression cases (hazard ratio 0.98 [95% CI 0.46–2.07]), although CIs were very wide. A systematic review and meta-analysis of PA interventions reported reduced depression symptoms (standardised mean effect size 0.37 [95% CI 0.24–0.50] for supervised PA studies and 0.52 [95% CI 0.28–0.77] for unsupervised studies) [21], supported by a systematic review of exercise referral schemes, which also showed reduced clinical depression risk in the two studies reporting this outcome (pooled standardised mean difference −0.82 [95% CI −1.28 to −0.35]) [41]. Of note, most studies included in the reviews reported depression risk based on symptom scores rather than clinical diagnoses of depression; we extracted the latter from primary care records, which could underestimate depression risk, as it relies on patients presenting with symptoms and general (family) practitioners detecting and recording them. Our negative finding from primary care records is, however, consistent with our main trial outcomes, which showed no intervention effects on depressive symptoms at 12 months [23, 24] or 3–4 years [25].

Cohort studies support an association between PA levels and depression, with increased PA levels associated with reduced depression risk, with the majority of studies (25/30) in a systematic review reporting this, though no meta-analyses or forest plots were presented [42], so there is no overall effect estimate for direct comparison with our findings.

### Implications for clinicians, researchers, and policy makers

An important implication for both future clinical practice and policy is that primary care short-term pedometer-based walking interventions incorporating behaviour-change techniques can lead not only to long-term changes in PA levels but also to long-term beneficial health effects for adults and older adults. They could thus help to address the public health physical inactivity challenge and be part of the ‘call to activity’ for clinicians and patients [43]. This supports current guidance to promote pedometers alongside support for goal-setting, self-monitoring, and feedback [44] and suggests that policy makers should consider investment in this short-term primary care pedometer-based walking intervention because of its proven long-term health benefits. Our previous work has shown that sustained effects on PA levels were similar for postal and nurse-supported intervention groups [25] and that the postal route was more cost-effective [28]; this route therefore seems most promising to pursue for implementation.

Our demonstration of the widespread acceptance by trial participants for their primary care records to be accessed and the feasibility of using these data for evaluating long-term health outcomes is consistent with others’ findings [22, 35]. Our experience supports initiatives from funders such as the Medical Research Council and the National Institute for Health Research in the UK and other funders internationally, to encourage researchers undertaking trials to include options for longer-term data collection from routine records in their funding applications. We found only one other example of a PA trial that used routine primary care data to assess long-term health outcomes [15]. Using such outcomes provides objective evidence applying to all those randomised (not just those who complete trial follow-up), and by focusing on the clinical benefits, it also avoids the problems inherent in measuring change in PA levels in large numbers of subjects. Linking healthcare data to trial data can strengthen the evaluation and implementation of primary care–based interventions and should be more widely explored by researchers.

### Unanswered questions and future research

Observational studies strongly suggest that the dose-response association between PA levels and both all-cause mortality [45] and a range of chronic diseases [36] is nonlinear, with the greatest benefit appearing when changing from a sedentary lifestyle to low levels of activity and smaller additional benefits from higher levels of activity [36, 45]. Such data have influenced the newly published Physical Activity Guidelines for Americans [46]. Our data, based on RCT evidence, are important for silencing the sceptics who argue that the new guidelines lack high-quality evidence from RCTs [43]. It is too soon to conclude with any confidence that relatively modest changes in PA can achieve greater health benefits than predicted from cohort studies, but that is the hope. Observational studies, even those using objective PA measures, cannot provide direct evidence of what happens when individuals change their PA levels by modest amounts; what is needed is for more PA trials that have successfully increased PA levels to provide long-term follow-up of clinical outcomes.

### Conclusions

We showed significant reductions in both cardiovascular events and fractures for the PA intervention groups from two trials over a 4-year period, supporting and extending the benefits demonstrated by the long-term trial PA outcomes. However, our CIs are wide, indicating uncertainty regarding the exact magnitudes of the effects. We also confirmed the feasibility of using routine primary care data to provide robust long-term health outcomes for RCTs. Short-term 12-week pedometer-based walking interventions can have long-term positive health effects and should be used more widely to help address the public health physical inactivity challenge.

## Supporting information

S1 Fig Details of how events were counted in downloaded primary care data. (TIF)

S1 Text Protocol for extended follow-up and implementation research for PACE-UP trial including primary care data download. (DOC)

S2 Text CONSORT checklist for the PACE-UP trial 4-year primary care data outcomes paper. (DOCX)

S3 Text Protocol for extended follow-up of PACE-Lift trial including primary care data download. (DOC)

S4 Text CONSORT checklist for the PACE-Lift trial 4-year primary care data outcomes paper. (DOCX)

S5 Text Comparison of physical activity levels from our study with cohort studies for estimating cardiovascular and diabetes relative risks. (DOCX)

S1 Table Coefficients for Cox regression and negative binomial models. (DOCX)

S2 Table Details of physical activity intervention studies with health outcomes. (DOCX)

## Abbreviations

ARR: absolute risk reduction
BMI: body mass index
CI: confidence interval
HADS: hospital anxiety and depression scale
IQR: interquartile range
MET: metabolic equivalent
MVPA: moderate-to-vigorous physical activity
NNT: number needed to treat
NNTH: number needed to harm
PA: physical activity
RCT: randomised controlled trial
REC: Research Ethics Committee
