# Supplementary figures and images for: Effect of pedometer-based walking interventions on long-term health outcomes: Prospective 4-year follow-up of two randomised controlled trials using routine primary care data
Source: PLoS Med. 2019 Jun 25;16(6):e1002836. doi: 10.1371/journal.pmed.1002836 (PMC6592516; doi:10.1371/journal.pmed.1002836)

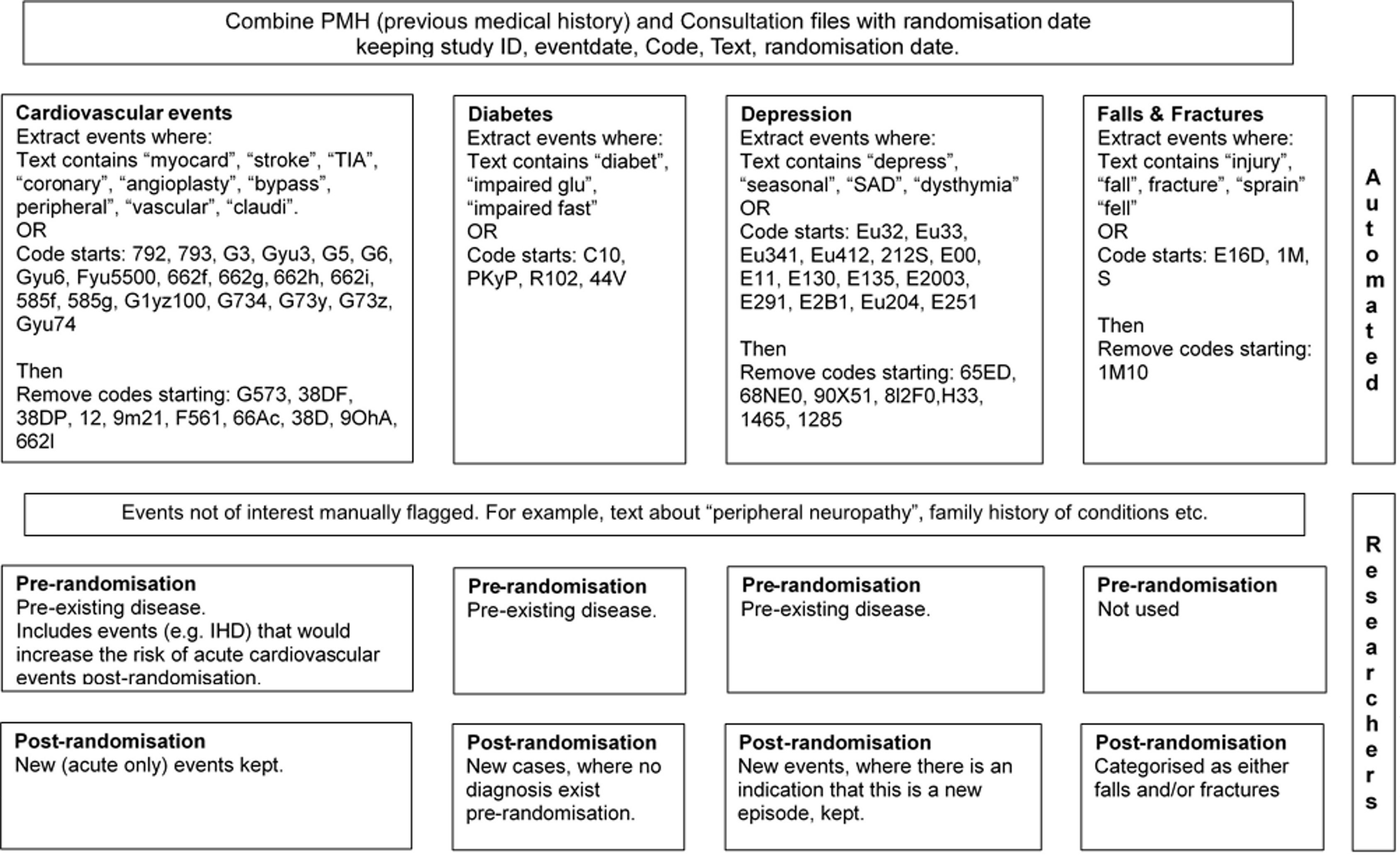

Supplement: S1 Fig — (TIF) [file pmed.1002836.s001.tif]
