# Supplementary material for: Effect of pedometer-based walking interventions on long-term health outcomes: Prospective 4-year follow-up of two randomised controlled trials using routine primary care data
Source: PLoS Med. 2019 Jun 25;16(6):e1002836. doi: 10.1371/journal.pmed.1002836 (PMC6592516; doi:10.1371/journal.pmed.1002836)
Supplement: S1 Text — (DOC) [file pmed.1002836.s002.doc]

**Trial protocol and further implementation research**

**Randomised controlled trial of a pedometer-based walking intervention with and without practice nurse support in primary care patients aged 45-75 years**

**trial (Pedometer And Consultation Evaluation - Up)**

**Unique trial identifier: ISRCTN98538934**

**Protocol version number 7.0**

**Proposed starting date: 01/03/2012 Proposed duration: 75 months**

**Proposed ending date: 31/05/2018**

**Principal Investigator:**

Dr Tess Harris, Senior Lecturer in Primary Care & General Practitioner, Population Health Sciences & Education, St George’s University of London, SW17 ORE. Tel: 0208 725 2791 email: [tharris@sgul.ac.uk](mailto:tharris@sgul.ac.uk)

**Co-applicants:**

Professor Derek Cook, Professor of Epidemiology, Population Health Sciences & Education, St George’s University of London, SW17 ORE. Tel: 0208 725 5490. Email: [d.cook@sgul.ac.uk](mailto:d.cook@sgul.ac.uk)

Mrs Sally Kerry, Reader in Medical Statistics, Pragmatic Clinical Trials Unit, Barts & The London School of Medicine & Dentistry. E1 2AB. Tel: 0207 882 7208 Email: [s.m.kerry@qmul.ac.uk](mailto:s.m.kerry@qmul.ac.uk)

Professor Christina Victor, Professor of Gerontology and Health Services Research, Brunel University, London UB8 3PH. Tel: 01895 268730. Email [Christina.victor@brunel.ac.uk](mailto:Christina.victor@brunel.ac.uk)

Dr Sunil Shah, Senior Lecturer in Public Health, Population Health Sciences & Education, St George’s University of London, SW17 ORE. Tel: 0208 725 0066. Email [sushah@sgul.ac.uk](mailto:sushah@sgul.ac.uk)

Professor Steve Iliffe, Professor of Primary Care for Older People, University College, London, NW3 2PF. Tel: 0207 8302393. Email: [s.iliffe@ucl.ac.uk](mailto:s.iliffe@ucl.ac.uk)

Dr Michael Ussher, Reader in Health Psychology & consultant to this project on pedometer and physical activity aspects of the intervention. Tel 0208 725 5605 [mussher@sgul.ac.uk](mailto:mussher@sgul.ac.uk)

Professor Julia Fox-Rushby, Professor of Health Economics, Health Economics Research Group, Brunel University, London UB8 3PH. Tel: 01895 266864 [julia.fox-rushby@brunel.ac.uk](mailto:julia.fox-rushby@brunel.ac.uk)

Dr Ulf Ekelund, Programme Leader Physical Activity Epidemiology, MRC Epidemiology Unit, University of Cambridge, CB2 OQQ. Tel: 0123 769208. Email: [ulf.ekelund@mrc-epid.cam.ac.uk](mailto:ulf.ekelund@mrc-epid.cam.ac.uk) and Department of Sport Medicine, Norwegian School of Sport Sciences, PO Box 4014, Oslo 0806, Norway [ulf.ekelund@nih.no](mailto:ulf.ekelund@nih.no)

Professor Peter Whincup, Professor of Epidemiology, Public Health Sciences & Epidemiology, St George’s University of London, SW17 ORE. Tel: 0208 725 5577. Email: [p.whincup@sgul.ac.uk](mailto:p.whincup@sgul.ac.uk)

**Collaborators:**

Dr David Finch, GP & Joint Medical Director NHS South West London Cluster,120, The Broadway, Wimbledon, London SW19 1RH. Tel: 0208 251 0158 email: dfinch@nhs.net

Dr Judith Ibison, Senior Lecturer in Primary Care & General Practitioner, Population Health Sciences & Education, St George’s University of London, SW17 ORE. Tel: 0208 725 2798 email: [jibison@sgul.ac.uk](mailto:jibison@sgul.ac.uk)

Dr Stephen DeWilde, Senior Lecturer in Primary Care & General Practitioner, Population Health Sciences & Education, St George’s University of London, SW17 ORE. email: [sdewilde@sgul.ac.uk](mailto:sdewilde@sgul.ac.uk)

Mrs Liz Limb, Statistician, Population Health Sciences & Education, St George’s University of London, SW17 ORE. email: [elimb@sgul.ac.uk](mailto:elimb@sgul.ac.uk)

Dr Lee David, General Practitioner & Educational Company Director, 10 Minute CBT, Tel: 0845 0948599. Email [lee.david@10minutecbt.co.uk](mailto:lee.david@10minutecbt.co.uk) website: [www.10minuteCBT.co.uk](http://www.10minuteCBT.co.uk/)

**Clinical trial unit support:**

Pragmatic clinical trials unit (PCTU), Barts & The London School of Medicine & Dentistry, E1 2AB.

Tel: 0207 882 7208.

**Sponsor:**

St George’s University of London, Dr Deborah McCartney Clinical Research Governance Officer, Cranmer Terrace Tooting, London, SW17 ORE. Tel: 0208 7256073. Email [dmccartn@sgul.ac.uk](mailto:dmccartn@sgul.ac.uk)

**Funders:**

Original trial funder

NIHR Health Technology Assessment Programme, NETSCC, Alpha House, University of Southampton Science Park, Southampton, SO16 7NS. Tel: 023 8059 5586 email [hta@hta.ac.uk](mailto:hta@hta.ac.uk)

Implementation research funders

1. Collaborative Leadership in Applied Health Research and Care – South London,

Health Service and Population Research Department,Institute of Psychiatry, Psychology & Neuroscience, King's College London, De Crespigny Park, London SE5 8AF. [clahrcsouthlondon@kcl.ac.uk](mailto:michele.harris-tafri@kcl.ac.uk)

1. St George’s University of London, Strategic Development Funding,

St George’s University of London, Cranmer terrace, Tooting, London, SW17 0RE

**Signature Page**

**Chief Investigator:
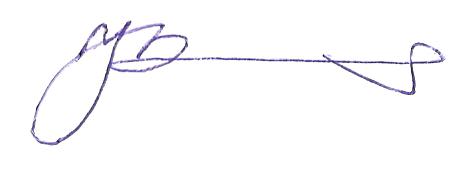
**

Dr Tess Harris _________________________________ Date 31/03/2017

**Trial statistician:**
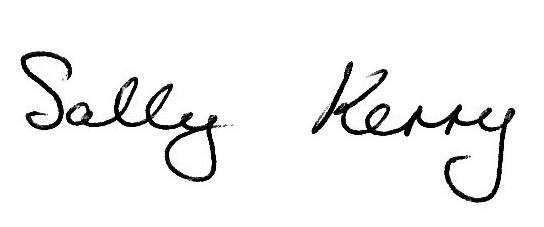


Mrs Sally Kerry _________________________________ Date 31/03/2017

**Contents Page**

1. **Summary** 6
2. **Background and rationale** 6
3. **Study objectives and purpose** 11
4. **Study design** 12

*Description of type of trial*

*Schematic diagram of trial design, procedures and stages*

*Primary and secondary endpoints / outcomes*

*Methods and timing for assessing, recording and analysis of outcomes*

*Measures taken to minimise / avoid bias*

1. **Participant selection** 16

*Participant inclusion criteria*

*Participant exclusion criteria*

*Participant recruitment and number of participants*

*Participant selection for the qualitative evaluation*

*Participant withdrawal*

1. **Study procedures** 17

*Informed consent procedures*

*Randomisation procedures*

*Baseline, 3 month & 12 month assessments*

*Procedure for control group*

*Nature of the intervention & components of the complex intervention*

*Procedure for the pedometer plus written instructions group.*

*Procedure for the pedometer plus practice nurse support group*

*Procedure for qualitative study*

*Procedure for health economic evaluation*

*End of study definition*

*Participant withdrawal*

*Data collection and follow-up for withdrawn subjects*

1. **Safety** 21

*Risks*

*Adverse events & serious adverse events*

*Causality*

*Notification and reporting of adverse events and serious adverse events*

*Retrospective data collection on adverse events*

*Monitoring*

*Annual safety reporting*

1. **Statistics** 23

*Sample size*

*Anticipated recruitment*

*Statistical analysis*

*Stopping rules*

*Procedure for accounting for missing, unused and unexpected data*

1. **Data handling and record keeping** 24

*Confidentiality & data protection*

*Maintenance of study documents*

*Record retention & archiving*

1. **Ethics, compliance & clinical governance** 25

*Ethical considerations*

*Compliance*

*Clinical governance issues*

1. **Monitoring procedures** 26

*Summary monitoring plan*

*Trial oversight committees: Trial Management Group, Trial Steering Committee*

*Before the start of recruitment*

*During the trial*

*At the end of the trial*

*Audit and inspection*

1. **Finance and insurance** 27

*Contracts & Financial Management*

*Insurance and indemnity arrangements*

1. **Publication and dissemination policy** 27
2. **Further 3 year follow-up of the PACE-UP trial cohort** 28
3. **PACE-UP trial implementation work – “PACE-UP Next Steps”** 29
4. **References** 33

1. **Appendices** 38

*Table 1**: Behaviour change techniques used in consultations with the practice nurse Table 2: Behaviour change techniques included in the PACE-UP trial Handbook and diary*

1. **Summary**

**Background:** Physical activity is vital for health, yet most adults and older adults are inactive and do not achieve the recommended 30 minutes of moderate intensity activity on 5 or more days weekly. Moderate intensity activity makes you warm and increases breathing and heart rate, but should allow conversation. Adults’ most common physical activity is walking, light intensity if strolling, moderate if brisker (5km/hr). Pedometers measure step-count and can increase walking, but most trials have had short term outcomes, have not separated out pedometer effects from other support and have reported only step-counts, not time spent at different physical activity intensities. This trial will investigate whether inactive patients aged 45-75 years can increase their physical activity by being given a pedometer with a diary and written guidelines and whether additional individual, tailored, support from a practice nurse increases any benefits.

**Design:** Primary care based 3-arm randomized controlled trial with 12 month follow-up and health economic & qualitative evaluations.

*Participants:* 993 inactive patients aged 45-75 years with no contraindications to increasing their moderate intensity PA will be recruited by postal invitation from 6 South West London general practices and randomly allocated into three groups. All participants will have their PA assessed objectively (step-count & time spent at different PA intensities) for 7 days at baseline, 3 months and 12 months by wearing an accelerometer(Actigraph GT3X+ Manufacturing Technology Inc) and a blinded pedometer, which give no patient feedback. They will also complete questionnaires and have anthropometric assessments.

*Intervention:* One intervention group (pedometer alone) will be posted out a pedometer (Yamax Digi-Walker SW-200) diary and written instructions for a 12-week pedometer-based walking programme, based on their own baseline blinded pedometer step-count. The second intervention group (pedometer plus support) will receive a pedometer and diary and three individually tailored PA consultations with a practice nurse. They will be supported to follow a 12-week pedometer-based walking programme, using strategies such as self-monitoring, goal-setting, boosting motivation and anticipation of set-backs. The control group will continue usual PA.

*Main outcome measures in all groups after 12 months:*

1. Change in average daily step-count (primary outcome measure, by accelerometry)
2. Change in sedentary time and time spent in at least moderate intensity PA weekly (by accelerometry)
3. Cost-effectiveness. Incremental cost of the intervention to the NHS and incremental cost per change in step-count and per quality adjusted life year.
4. Acceptability of the interventions.
5. **Background and rationale**

*Why is PA important and what are the guidelines for PA levels for adults and older adults?*

Physical inactivity is extremely detrimental to health. It can lead to many important negative health consequences and to increased overall mortality^1;2^. Adults, including older adults, are advised to be active daily and over a week their activity should add up to at least 150 minutes (2 ½ hours) of moderate intensity activity in bouts of 10 minutes or more, for optimum health benefits. One effective way to achieve this is to do 30 minutes activity on at least 5 days a week^1-3^. Moderate intensity PA makes you warm and increases breathing and heart rate, but should allow talking. Regular walking is the commonest PA of adults and older adults and is specifically promoted as a near perfect exercise, even walking at a moderate pace of 3 miles/hr (5km/hr) expends sufficient energy to qualify as moderate intensity PA, but with very low risk of harm^4^. Faster walking speeds are associated with reduced mortality^5^. UK public health policy emphasises helping adults and older adults to increase their PA, particularly through walking^6-8^. Both adults and older adults are also advised to minimise the amount of time spent being sedentary (sitting) for extended periods^1^.

*How much PA do adults and older adults do?*

Amongst adults aged 16 and over in England, 39% of men and 29% of women self-reported achieving the recommended PA levels. However, PA levels decrease considerably with age and only 20% and 17% of men and women aged 60-74 respectively reported achieving these levels^9^ despite most of those older people not walking being able to do so^10^*.* Time spent being sedentary is also an important independent disease risk factor^2^; this increases with age from 45 years^9^. As well as variations by age and gender there are also differences by socioeconomic status and ethnic group. Both men and women from lower socioeconomic groups report significantly lower PA levels than those from higher groups^9^. Men and women from Indian, Pakistani, Bangladeshi and Chinese ethnic groups are significantly less likely to achieve recommended PA levels, whilst other groups (Black Caribbean, Black African and Irish) are not significantly different from the general population^11^. Walking is the main physical activity reported by adults and older adults, but since it is unreliably recalled^12^, surveys may overestimate PA levels. Objective measurement of PA levels using accelerometers in a sub-sample of the Health Survey for England found that only 5% of men and 4% of women aged 35-64 years and 5% men and 0% of women aged 65 or more achieved the recommended PA levels, a fraction of those self-reporting achieving them^9^.

*Summary of benefits and risks of increasing PA.*

*What are the benefits for increasing physical activity levels?* The Chief Medical Officers from the four home countries recently published a report on physical activity for health, which has drawn upon recent international large scale reviews of the evidence on the impact of physical activity and its relationship to health across the lifecourse. The following benefits for adults and older adults are described: reduced mortality; a reduced risk of over 20 diseases and conditions (including cardiovascular disease, diabetes, obesity, osteoporosis, several cancers, depression, dementia); reduced falls risk; and improved function, quality of life and emotional well-being^1^. Physical inactivity is the fourth leading risk factor for global mortality (accounting for 6% of deaths globally). This follows high blood pressure (13%), tobacco use (9%) and high blood glucose (6%), with obesity and overweight accounting for 5% of deaths^13^. Physical inactivity is fourth on the list, but it influences most of the other causes. These effects are of major importance to both individuals and society, with the annual direct cost of physical inactivity to the NHS across the UK recently estimated at £1.06 billion^1^ based on five conditions specifically linked to inactivity. However, this figure represents a conservative estimate, as it excludes the cost of other diseases and health problems that affect many older people^1^. UK public health policy now has an emphasis on helping adults and older adults to increase their physical activity, particularly walking^6;7;14^.

*What are the risks from increasing physical activity?* Whilst there are risks for adults and older adults associated with regular physical activity, the risks of a sedentary lifestyle far exceed them^2;15;16^. Both the intensity of physical activity and its regularity need consideration when assessing risk. Violent, unaccustomed exercise is associated with a higher risk of myocardial infarction and death in the following 24 hours, but regular, vigorous exercise protects against such events^17^. Low intensity physical activity has the lowest injury risk, but moderate intensity physical activity has a better risk-to-benefit ratio and should be the goal for inactive adults and older adults^16^. Moderate intensity physical activity carries a low risk of injury^18^, the commonest adverse events are musculoskeletal injury or falls^19^. Walking appears to be very low risk and has been described as a near perfect exercise^4^. Screening all participants before taking part in physical activity programmes is no longer advocated, as there is a very low degree of risk for light to moderate intensity physical activity^2;20^. Ory et al reviewed adverse events from 11 diverse physical activity interventions including older people and patients with heart disease. Numerous adverse events occurred in sedentary, chronically ill, older populations, but very few were attributed to activity interventions. No serious study-related adverse events were reported, but some minor ones were (mainly musculoskeletal injuries)^20^. A more recent trial of a primary care nurse intervention in women aged 40-74 years, showed a small but significant increase in falls and injuries^21^. This trial advised 30 minutes of brisk walking five days per week. An important safety feature of our study is that baseline physical activity is recorded objectively on all participants, enabling individualised goals starting from the participant’s own baseline. This is in line with advice that before increasing physical activity, older adults in particular should have risk management strategies for prevention of activity-related injuries; the most important being to start with low intensity physical activity and increase intensity gradually, the “start-low-and-go-slow” approach^15;16^.

*How can adults and older adults increase their PA levels?*

A Cochrane systematic review of 17 RCTs of PA interventions reported moderate positive short-term effects in middle age (only 4 studies included older people) but findings were limited by most studies using self-report measures in motivated volunteers^22^. Effective interventions for increasing PA explored factors associated with behavioural change, including beliefs about costs and benefits of PA^23^. The American College of Sports Medicine updated position stand on prescribing exercise concluded that exercise programs in diverse populations have been effective in promoting short-term increases in physical activity when they are based on health behaviour theoretical constructs, are individually tailored and use behavioural strategies such as goal setting, social support, reinforcement, problem solving and relapse prevention^2^. A critical review and a best practices statement on older peoples’ PA interventions advised home rather than gym-based programmes and cognitive behavioural strategies (e.g. goal-setting, self-monitoring, feedback, self-efficacy, support, relapse prevention training) rather than health education alone^16;24^. These behaviour change techniques are emphasised in the NHS Health Trainer Handbook, based on evidence from a range of psychological methods and intended for NHS behaviour change programmes, with local adaptation^25^. Starting low, but gradually increasing to moderate intensity is promoted as best practice, with advice to incorporate interventions into the daily routine, e.g. walking and to monitor intensity for progression^16^. A recent systematic review of interventions to promote walking concluded that interventions tailored to people’s needs, targeted at the most sedentary or at those most motivated to change and delivered at the level of the individual or household can encourage people to walk more, although the sustainability, generalisability and clinical benefits of many of these approaches are uncertain^26^.

*What is the evidence that pedometers can help adults and older adults to increase their PA levels?*

Pedometers are small, relatively inexpensive devices, worn at the hip, that provide direct feedback on PA frequency, the number of steps walked per day (step-counts). In 2006 the National Institute of Health and Clinical excellence (NICE) produced guidance on the use of pedometers to increase the population’s physical activity levels^27^. They used stringent inclusion criteria and included evidence from only four quality randomised controlled trials involving different target groups^28-31^. They concluded that the evidence for pedometer based interventions in increasing PA levels in the adult population was equivocal in both the short and longer term. Specifically, a lack of evidence was found on the effect of pedometers on overall PA levels, as an adjunct to other interventions, and on long term outcomes (no evidence was found which examined effectiveness at one year). They recommended that pedometer based interventions should only be endorsed if part of a properly designed and controlled research study to determine their effectiveness^27^. Two subsequent systematic reviews and meta-analyses have been published. The first was based on 26 studies (8 RCTs and 18 observational studies) and found pedometer users increased steps/day by 2491(1098-3885) and PA levels by 27%. There were also significant reductions in body mass index and systolic blood pressure^32^. The most recent review was based on 32 studies and found that the combined effect size was 0.68 (95% CI 0.55-0.81) translating to an average increase of 2000 steps in the intervention group, indicating that pedometers are useful motivational tools to increase physical activity^33^. Both reviews found that setting a step-goal and using a step diary were key motivational factors for increasing physical activity^32;33^. Several limitations of the reviews were recognised. Firstly, study sizes were relatively small (the largest RCT had 330 participants, but the majority had less than 50) and interventions were of relatively short duration (mainly 12 weeks or less), so whether changes were durable over the long term was undetermined. Secondly, because many studies included several components (eg pedometer and counselling) the independent contribution of these components was difficult to establish. Finally, the inclusion of older people and men in studies included in the reviews was very limited^32;33^. Some more recent studies have focused on older people, had larger samples and attempted to isolate the pedometer’s effects. An American trial in 147 older people showed an increase of 1320 steps/day at 12 weeks^34^, but provided no longer term follow-up. A Scottish trial of 210 older women found that physical activity was increased at 3 months by a pedometer plus behaviour change intervention (BCI), but the provision of pedometers yielded no additional benefit to the BCI apart from reducing drop-outs, and increased physical activity was not sustained by 6 months^35^. Two other recent trials in high risk groups (cardiac patients n=65, and impaired glucose tolerance n=87) have shown sustained increases in step-count at 12 months^36;37^.

*What is the evidence about different step goals with pedometer interventions and how do these relate to physical activity recommendations for adults?*

Pedometer users who were given a step goal were significantly more likely to increase their steps than those without a step goal. However, no difference was found between the different step-goals set^32^. Broadly studies have set goals based on either a fixed target (eg 10,000 steps/day)^38;39^ or on taking an individual’s baseline target and advising incremental increases, either as a percentage increase or by a fixed number of extra steps per day. Those advocating a percentage increase have tended to suggest 5% increase per week^34^, 10% biweekly^40^ or 20% monthly^35^. Those advocating a fixed number of extra steps per day have tried to develop step-based guidelines that fit with existing evidence based physical activity guidelines with their emphasis on 30 minutes of at least moderate intensity physical activity on 5 or more days per week^41^. There is evidence that despite some individual variation, moderate intensity walking appears approximately equal to at least 100 steps per minute^41;42^. If this is multiplied by 30 minutes (typical daily recommendation) it produces a minimum of 3000 steps per day, that is best used as a guiding value, but these steps need to be taken over and above habitual activity (or baseline). Several studies have advocated adding in 3000 steps/day on most days per week, either increasing by this amount from the beginning^36^ or by increasing in an incremental manner (starting with an extra 1500 steps/day initially and gradually increasing)^43;44^ or increasing by 500 steps/day every 2 weeks^37^. All of these studies that have advised adding 3000 steps/day to baseline have produced significant improvements in step-counts at 3 months and two measured outcomes at 12 months and showed sustained improvements in step-counts^36;37^, waist circumference^36^ and fasting glucose levels^37^. Although there is no evidence at present to inform a moderate intensity cadence (steps/minute) in older adults, Tudor-Locke at al advocate using the adult cadence of 100 steps/minute in older adults (whilst recognising that this may be unobtainable for some individuals) and advising that the 30 minutes can be broken down into bouts of at least 10 minutes^45^. This is the model used in a primary care trial of a walking intervention in 41 older people which found significant increases in step-counts from baseline to week 12, maintained at week 24 and advised a larger trial to confirm findings^46;47^.

*Could accelerometers be useful in a study designed to increase walking?*

Accelerometers are also small activity monitors, worn in a similar way to pedometers, much more expensive, but able to provide a time-stamped record of both PA frequency (step-counts) and PA intensity (activity counts). They require computer analysis and therefore give no direct feedback to participants. They therefore function as blinded pedometers in objectively measuring baseline and outcome PA data, but have the additional advantage of being able to provide objective data on time spent in different levels of PA intensity, particularly time spent in at least moderate intensity activity and time spent in sedentary activity, two important public health outcomes. Pedometer studies that do not use accelerometers have relied on self-report measures to assess these outcomes. Accelerometers have been shown to be valid and acceptable to adults^9;48^ and older adults^9;49;50^. Although accelerometers measure step-count and there is a strong correlation between step-counts measured on accelerometers and pedometers^51;52^ pedometers usually underestimate step-counts relative to accelerometers, particularly at lower walking speeds, and one instrument’s step-counts may not easily be substituted for the other at an individual level^52^. Thus, although we will use the accelerometer as the main outcome measure to assess change in both step-count and time spent in different physical activity intensities, we will use the blinded pedometer to set the individual step-count targets for individuals.

*What is known about the cost-effectiveness of promoting PA through pedometer programmes?*

To our knowledge, no study has examined the cost-effectiveness of pedometer based interventions in the UK. Evidence that exists on economic evaluation of PA interventions tends to focus on behavioural and environmental interventions^53-55^. Recent systematic reviews that considered the economic outcomes of pedometer based interventions found no evidence ^56;57^.This has partly been attributed to insufficient data on the effect of pedometers on PA^58^. However, one Australian study has recently modelled the cost-effectiveness of a community programme to encourage use of pedometers as a motivational tool to increase PA in an Australian cohort of people aged >14 years^59^. Using disability adjusted life years as the main outcome measures, the authors found the intervention to dominate the alternative of ‘do-nothing’ and be cost-saving. The different context for this analysis however, makes it difficult to relate this finding to decision making in the UK. We are also aware that the cost-effectiveness of any intervention depends on participation, and our own research^60^ shows that the demand for sports and exercise is negatively associated with time (travel time) and money cost that vary by participation (e.g. entrance charge) and positively associated with money cost that does not vary by participation (e.g. membership fee) holding socio-economic and demographic factors constant.

*What is primary care’s role in promoting PA?*

Primary care is accessible and offers continuity of care for adults and older adults, with many chronic diseases being an indication for increasing PA. Health professional PA advice in consultations is individually tailored^61^ and has greater impact than other PA advice^62^. Primary care nurses have been shown to be effective at increasing physical activity, particularly walking, in this age group^21^. PA promotion by other routes for older adults in particular is unlikely to be as effective^63^. Recent guidance on prescribing exercise in primary care reinforces the importance of follow up to chart progress, set goals, solve problems and identify and use social support^64^ this will be an important feature of the nurse physical activity consultations in this trial. NICE Public Health Intervention Guidance on PA found that brief interventions in primary care are cost-effective, these varied from basic advice to offering extended and individually tailored consultations to identify and motivate change in physical activity behaviour. NICE therefore recommends that all primary care practitioners should take the opportunity, whenever possible, to identify inactive adults and advise them to aim for 30 minutes of moderate intensity activity on 5 days of the week or more^27^. New NHS Health Checks rolled out by 2013, include adults up to age 74 & incorporate advice on increasing PA, often by primary care nurses^65^. Evaluation of the UK Step-O-Meter Programme, delivering pedometers through primary care, in combination with physical activity consultations, showed self-reported PA increases, but advised investigation with a RCT design^43^. Two small trials have assessed the effectiveness of pedometers in combination with physical activity consultations: one through a community based programme showed a significant effect on step-counts at 12 weeks in 79 middle aged adults^44^; the other showed a significant effect on step-counts at 12 weeks, maintained at 24 weeks in 41 older primary care patients and called for a further, larger primary care trial^46;47^.

*Policy relevance.* UK public health policy emphasises helping adults and older adults to increase their physical activity, particularly walking^6;7;14^. MoreActive4Life is the physical activity strand of the Change4Life programme, a society wide approach to encouraging people to eat well, move more and live longer^8^. New NHS Health Checks being rolled out by 2013, include adults up to age 74 & incorporate advice on increasing physical activity, often by primary care nurses^65^. These directives underline the importance of getting adults and older adults to be more active and confirm the timeliness of our research for examining effective ways to achieve this.

*Theory on which the intervention is based and relevant pilot and preparatory work*

The pedometer-based intervention is based on previous work cited above showing that pedometers are effective at increasing steps, but extending this to ensure that the study covers older adults, men, has a 12 month follow-up and is designed to examine separately the role of the pedometer and the role of support. The practice nurse physical activity consultations will use behaviour change techniques (e.g. goal-setting, self-monitoring, feedback, building self-efficacy, encouraging social support, relapse prevention training etc). These techniques are emphasised in the NHS Health Trainer Handbook, based on evidence from a range of psychological methods and intended for NHS behaviour change programmes, with local adaptation^25^. We have adapted the NHS health trainer handbook for use in this trial by the nurses to focus specifically on physical activity and using pedometers. The behavioural change techniques to be used have been classified according to Michie’s refined taxonomy of behaviour change techniques for physical activity interventions^66^. (See Tables 1 and 2 for more details about behaviour change techniques used in nurse consultations and in PACE-UP handbook and diary respectively). Relevant pilot and preparatory work includes observational work using pedometers and accelerometers in a primary care setting^49^ and a trial developing the physical activity consultations and pedometer based walking intervention in a study with older primary care patients (PACE-Lift trial funded by Research for Patient Benefit ISRCTN42122561^67^).

*Population to be studied.* The population to be studied will be inactive adults and older adults aged between 45-74 years who are registered with six different general practices in South West London, who can walk outside the home and who have no contraindications to increasing their moderate intensity physical activity levels. We are using a single item validated questionnaire measure of self-reported physical activity^21^ to select inactive adults. Those reporting achieving the Chief Medical Officers’ guidelines of at least 150 minutes of at least moderate intenstiy physical activity weekly^1^ will be excluded.

*Rationale*

These findings suggest that there is a need for a large, adequately powered study based in primary care to test the effect of a pedometer based walking intervention, with and without nurse physical activity consultations to support the intervention. The study should include follow-up to one year and ensure that adequate numbers of men and older adults as well as individuals from diverse socio-economic and ethnic backgrounds are included. For greatest effect the intervention should use step-goals and diaries and the physical activity consultations should be based on cognitive behavioural strategies, such as those used in the NHS Health Trainer Handbook^25^. In order to objectively test the effectiveness of the intervention on important public health outcomes, such as time spent in sedentary activity and time spent in at least moderate intensity activity, accelerometer measurement of outcomes should be included. The study should include a qualitative assessment to explore the intervention’s acceptability and reasons for dropout and durability of effects. An economic evaluation should be performed alongside the trial and the costs and benefits of the alternatives should be modelled beyond the end of the trial.

*Statement of trial conduct*

The trial will be conducted in compliance with the protocol, GCP and the relevant regulatory requirements.

1. **Study objectives and purpose**

**Research objectives**

**Primary objectives** - in inactive adults aged 45-75 years:

1. To determine whether or not the simple provision by post of pedometers plus written instructions for a pedometer-based walking programme, can promote an increase in step-counts and time spent in at least moderate PA compared to control at 12 months (pedometer vs control);
2. To determine whether or not tailored support from practice nurse PA consultations combined with a pedometer based walking programme can promote an increase in step-counts and time spent in at least moderate PA compared to control at 12 months (pedometer plus nurse support vs control);
3. To determine whether or not the addition of tailored support from practice nurse PA consultations to the pedometer based walking programme alone, can promote a worthwhile increase in any effect on step-counts and time spent in at least moderate PA at 12 months (pedometer plus nurse support vs pedometer);
4. To determine the cost-effectiveness of these alternative approaches to increasing PA levels at both 12 months and from a life time perspective from the viewpoint of the NHS, personal social services and participants;

**Secondary objectives:**

1. To determine whether age groups, gender, taking part as a couple, socio-economic-demographic sub-groups, level of baseline PA and health status modify the effect of the intervention on increasing PA levels at 3 months and 12 months;
2. To explore the intervention’s acceptability to practice nurses and inactive adults, reasons for dropout and durability of effects, by qualitative interviews after 12 month follow-up and focus groups on study completion;
3. To assess the fidelity and quality of intervention implementation over time by evaluation of patient diary step-count goals and recorded step-counts for both intervention groups at 3 month assessment and number and timing of recorded practice nurse contacts for nurse support group.

**4.** **Study design**

*Description of type of trial*

3-arm primary care based randomised controlled trial with 12 month follow-up comparing the following: a control group (usual physical activity); pedometer & written instructions; pedometer & support (written instructions and brief individually tailored PA consultations with a practice nurse). Qualitative, process and economic evaluations will run parallel to the trial.

*Schematic diagram of trial design, procedures and stages*: See Consort flow diagram (page 14).

*Primary and secondary endpoints*

*Primary outcome*

Change in average daily step-count, measured over 7 days, between baseline and 12 months assessed objectively by accelerometry (Actigraph GT3X+ Manufacturing Technology Inc, Fl USA).

*Secondary outcomes (other main outcomes)*

Change in time spent in i) at least moderate intensity physical activity and ii) sedentary physical activity between baseline and 12 months, measured over 7 days by accelerometry.

Change in average daily step-count, time spent in at least moderate physical activity and time spent sedentary measured over 7 days, between baseline and 3 months assessed objectively by accelerometry

Cost-effectiveness. Incremental cost of the intervention to the NHS and incremental cost per change in step-count and per quality adjusted life year.

Acceptability of the interventions (qualitative interviews and focus groups)

*Other outcome measures:*

1. change in self-reported physical activity from the questionnaire (General Practice Physical Activity Questionnaire (GPPAQ) International Physical Activity Questionnaire (IPAQ)
2. change in other patient reported outcomes from the questionnaire (exercise self-efficacy, anxiety, depression, perceived health status including mood and pain (EuroQol 5D [EQ-5D])
3. change in anthropometric measurements (weight, body mass index, waist circumference, body fat, bio-impedence (Tanita scales)
4. adverse outcomes (data on falls, injuries, major cardiovascular disease events and deaths will be collected as part of safety monitoring for the trial, through participant and nurse reporting, questionnaires at 3 and 12 months and primary care records after 12 month follow-up
5. health service use - number of and diagnoses for all primary care consultations during the 12 months of the trial, as well as any out of hours, A&E or in-patient attendances related to falls, injuries, fractures etc from computerised primary care records at the end of the study given participants’ consent.

*Qualitative outcomes*

There will be a range of outcomes from qualitative interviews and focus groups for both participants and practice nurses involved in implementing the intervention. We will gain an in-depth understanding of the acceptability and challenges with the interventions for participants and practice nurses, as well as valuable insights into the factors influencing why people opt not to participate in the intervention.

*Methods and timing for assessing, recording and analysis of outcomes:* The RAs will conduct all the primary outcome assessments at 12 months. The RAs will arrange postal collection of questionnaire and accelerometer data at 3 months. Data entry and recording of accelerometer data will occur as soon as each accelerometer is returned, when data will be downloaded. Data entry of questionnaire data will occur as soon as possible after data collection at each time period. Analysis of outcome data will occur when data on all participants is collected.

*Measures taken to minimise / avoid bias*

*Randomisation:* Participants completing the baseline assessment (including returning the accelerometer with at least 5 complete days of recording ≥9 hours / 540 minutes per day) will be allocated to the trial groups by the RAs using the Kings College London Clinical Trials Unit (CTU) internet randomisation service to ensure independence of the allocation. Randomisation will be at household level to avoid couple contamination, which could occur if a couple were allocated to different arms. See section 6: Study Procedures for full details.

*Contamination:* Contamination could occur between partners in the same household, we will minimise this by ensuring that if both are recruited they are allocated to the same group. Contamination could also occur in the control group if they seek their own way of increasing their physical activity and particularly if they obtain and use their own pedometer. We will try to minimise this by ensuring that control participants know that they will receive a free, accurate pedometer after the 12 month follow-up and we will monitor this by asking about pedometer use in the 12 month assessment.

*Outcome assessment:* The RA will arrange all the outcome assessments at 3 months (postal) and 12 months (at the practice). Appointments for these will be booked in advance according to a protocol, taking into account holidays. The RA assessing outcomes will not be blinded to the participants’ intervention status for pragmatic reasons only; the study is funded to support only two RA to carry out recruitment and follow-up simultaneously at 3 practices each. If two RAs were required to cover each practice in order to carry out different assessments we would require further RA support for the trial. However, primary and secondary outcome measures (as above) are objectively measured by accelerometry and do not rely on assessor interpretation. Physical measurements will also be assessed objectively e.g. weight & body fat assessment. Patient reported outcomes (e.g. exercise self-efficacy, anxiety and depression, perceived health status including mood and pain, EuroQol 5D (EQ-5D)) will be assessed by validated self-report instruments, minimising researcher bias.

*Statistical analysis:* The senior statistician (SK) will analyse the data blind to the treatment allocation of the participants.

**CONSORT flow diagram showing participant flow through each stage of the randomized trial (enrolment, intervention allocation, follow-up & data analysis).**

**Assessed for eligibility**

Patients aged 45-74 years registered with 6 practices.

N=approximately 1700 patients per practice=10,200 total

**Excluded (pre-screening questionnaire)**

N=1020 (approx 10%). Not meeting inclusion criteria: living in care home, housebound, terminal illness, dementia, psychotic illness etc.

**Potentially Eligible**

N=9180 patients (5400 households)

Random samples sent information on study and screening questionnaire to assess activity levels until required number of patients randomised

.

**NOT ASSESSED BY SCREENING QUESTIONNAIRE**

N= 1800 (more if response rate to trial higher)

**Sent questionnaires**

***Assuming 20% response rate to trial invitation***

7300 patients sent screening questionnaire (fewer if response rate to trial higher)

**NOT ENROLLED**

1800 (approx 25%) either too fit or contraindications to increasing physical activity as assessed by screening questionnaire

4400 refuse to participate (80% of eligible)

**RANDOM ALLOCATION**

N=993

(Participants informed of allocation by telephone)

**ENROLLED**

N=total 1100 patients (approx 680 households)

**Pedometer + support** (n=331 patients)

Pedometer plus written instructions & 3 PA consultations with a practice nurse.

Individually tailored consultations at week 1, week 5, week 9. Patient led goal setting, problem solving, self-monitoring, SMART goals, motivational language, social support, relapse prevention. Step-count central to goal setting & feedback. Seen individually or as couples.

Contact at 6 & 9m (by phone, text or email as per patient preference) to check on safety outcomes, contact details & offer support & motivation.

**Control** (n=331 patients)

Usual physical activity.

Controls are instructed to continue with their usual physical activities.

Contact at 6 & 9m (by phone, text or email as per patient preference) to check on safety outcomes, contact details & offer support & motivation.

**Pedometer** (n=331 patients)

Pedometer plus written instructions for 12 week walking intervention, based on own baseline step-count, including written advice on maintenance, posted out to patients.

Contact at 6 & 9m (by phone, text or email as per patient preference) to check on safety outcomes, contact details & offer support & motivation.

**NOT RANDOMISED**

107 (10%) do not return accelerometer or ineligible after baseline assessment

**BASELINE ASSESSMENT**

Confirm eligibility, informed consent, questionnaires, anthropometric measures,

7 day accelerometer and blinded pedometer monitoring of usual PA levels

**FOLLOW-UP ASSESSMENTS 3 & 12 MONTHS**

3 month postal assessment. RA arranges time to post out accelerometer for 7 day monitoring and questionnaires (self-report PA and PROs).

12 month assessment at practice. RA posts out accelerometer to record PA for 7 days prior to assessment. 12 month follow up visit with RA: accelerometer monitoring, self-report PA levels, PROs & anthropometry. Offered PA consultation with nurse & pedometer at end of trial.

**FOLLOW-UP ASSESSMENTS 3 & 12 MONTHS**

3 month postal assessment. RA arranges time to post out accelerometer for 7 day monitoring and questionnaires (self-report PA and PROs).

12 month assessment at practice. RA posts out accelerometer to record PA for 7 days prior to assessment. 12 month follow up visit with RA: accelerometer monitoring, self-report PA levels, PROs & anthropometry. Offered physical activity consultation with nurse at end of trial.

**FOLLOW-UP ASSESSMENTS AT 3 & 12 MONTHS**

3 month postal assessment. RA arranges time to post out accelerometer for 7 day monitoring and questionnaires (self-report PA and PROs).

12 month assessment at practice. RA posts out accelerometer to record PA for 7 days prior to assessment. 12 month follow up visit with RA: accelerometer monitoring, self-report PA levels, PROs & anthropometry.

**DATA ANALYSIS**

Assume 15% attrition 12 months n= 281

**DATA ANALYSIS**

Assume 15% attrition 12 months n= 281

**DATA ANALYSIS**

Assume 15% attrition 12 months n= 281

**5. Participant selection**

**DATA ANALYSIS**

(Assume 10% attrition) 3months n=135, 12 months n=120

*Inclusion criteria for practices:* Inclusion criteria will be as follows: being a practice in one of the 6 PCTs in the South West London cluster with a combined list size >9,000; giving a commitment to participate over the duration of the study; having a practice nurse interested and with time to carry out the physical activity interventions and trial procedures; and the availability of a room for the research assistant to recruit participants and carry out baseline and follow-up assessments.

*Participant inclusion criteria:* Patients aged 45-74 years registered at one of the 6 general practices within the South West London cluster where the research is being undertaken, able to walk outside the home and with no contraindications to increasing their moderate intensity physical activity levels. Whilst patients aged 45-74 years will be searched for on practice lists for recruitment, it is recognised that some patients will be 75 years by the time they are recruited into the trial, therefore the age range for the trial will be 45-75 years.

*Participant exclusion criteria:* The commissioning brief advocated a focus on inactive adults, in order to maximise the benefits of the intervention to individuals and the NHS. We are therefore using a single item validated questionnaire measure of self-reported physical activity^21^ to select inactive adults. Those reporting achieving the Chief Medical Officers’ guidelines of at least 150 minutes of at least moderate intensity physical activity weekly^1^ will be excluded. Research assistants will telephone those wishing to take part who report on the questionnaire that they achieve this level of physical activity and ask them in more detail about their physical activity and exclude those who report achieving this level. We anticipate that there will be a few participants who will be found on subsequent baseline accelerometer assessment to be above this level of physical activity despite reporting usual lower physical activity levels (our previous pilot work suggests this number will be small as most people tend to overestimate their physical activity levels). They will not be excluded from the study, as this is a pragmatic trial and if this intervention were to be rolled out, exclusion would be on the basis of the screening question alone. Other exclusions: (from pilot study & from ProAct65+^68^): living in a residential or nursing home; housebound; ≥3 falls in previous year or ≥1 fall in previous year requiring medical attention; terminal illness; dementia or significant cognitive impairment (unable to follow simple instructions); registered blind; new onset chest pain, myocardial infarction, coronary artery bypass graft or angioplasty within the last 3 months; medical or psychiatric condition which the GP considers excludes the patient (e.g. acute systemic illness such as pneumonia, acute rheumatoid arthritis, unstable or acute heart failure, significant neurological disease or impairment, unable to move about independently, psychotic illness). Pregnant women will also be excluded from the trial.

*Recruitment of practices:* General practices will be recruited from the Primary Care Research Network Greater London (PCRN-GL). PCRN-GL will help us to identify potential participant practices within the South West London cluster of 6 PCTs (Wandsworth, Sutton, Merton, Croydon, Kingston, Richmond) who fit the above practice inclusion criteria. Approaches by mailed invitation, telephone contact with practice managers and personal contact with local GPs and practice nurses will all be used as necessary to identify practices who may be interested in taking part. For ease of recruitment for research assistants and managing the trial we will ideally select the 6 practices as pairs of practices from 3 PCTs. Once we have a list of potentially interested practices we will select 6 (ideally as pairs from 3 PCTs) to include a range of socio-demographic factors (including targeting some practices with high numbers of ethnic minority patients) and geographical circumstances based on practice postcode index of multiple deprivation (IMD) scores (at least 1 practice from each quintile). The IMD score includes factors such as distance to services, crime rates and road traffic accident rates, which could influence likelihood of outdoor physical activity, as well as material deprivation measures.

*Recruitment of participants:*

The number of patients aged 45-74 years will be recorded at each practice. Practice staff will search practice electronic primary health care records to identify patients aged 45-74, using Read codes supplied by researchers and local care home knowledge to exclude ineligible patients (as per exclusion criteria, see above). A list of potentially eligible patients will be created and ordered by household, with each household given a unique household identifier number. An Excel file with all identifying details removed will be taken to SGUL for random sampling (the file will just have EMIS or other practice database IDNO, Age, Group household (yes or no) household IDNO. This file will be used to create 4 random samples (A,B,C,D) and spares (X) of 400 individuals each (i.e.1600 individuals plus spares) using the random number generation feature in Stata. We want to select a maximum of 2 people per household and we are aiming to select couples. If a household with one individual is selected at random, then that individual is selected. If a household with two individuals is selected then one of the 2 is selected at random, if the other individual has an age difference of 15 years or less, then they will be selected too. If the age difference is greater than 15 years, only the first individual in that household will be selected. If a household has more than 2 individuals then one individual is selected at random. If there is a second individual in that household with an age difference of 15 years or less, they will also be selected, but if not then only the first individual in that household will be selected. Initially the first random sample containing 400 eligible patients will be selected at each practice and the list will be examined by practice GPs to ensure trial suitability. Patients in these households will then be mailed an individual invitation letter about the trial from the practice and a screening questionnaire to assess activity levels and any contraindications to increasing moderate intensity physical activity. (A single item validated questionnaire measure of self-reported PA^21^ will be used to select inactive adults, those reporting achieving the Chief Medical Officers’ recommended guidelines of at least 150 minutes of moderate intensity PA weekly will be excluded^1^). The participant information sheet giving more details about the study will be sent out with the patient invitation letter, it will make clear that if potential participants have any difficulties understanding, speaking or reading english they should bring a family member or friend with them to the appointment with the research assistant. The participant information sheet will be translated into different languages if practices indicate that this would be appropriate. The 400 individuals will be mailed out to in a staggered manner over the next 3-4 months to avoid overwhelming the research assistants. The other random samples of households (B, C, D) will be selected from the list at 3-4 monthly intervals until required numbers have been randomised (166 individuals in total to intervention and control groups in each practice). On the reply slip, those not wishing to take part in the trial will be asked about their willingness to fill in a questionnaire about their health, physical activity levels and reasons for not wanting to participate. Patients who agree to participate in the trial will be telephoned to arrange a baseline assessment at the practice with the research assistant. Two eligible people within a household will be invited together (or apart if they prefer). Eligibility will be confirmed and informed consent sought at the appointment with the research assistant.

*Participant selection for the qualitative evaluation:* This will run parallel to the trial and will focus upon three distinct groups. i) Trial ‘non-participants’ who agree to be interviewed, to explore factors influencing their decision not to participate. ii) Purposive samples of four groups of trial participants, after 12 month follow-up (including samples of those who did and did not increase their PA in each of the two intervention arms). The samples will reflect the range of socio-demographic characteristics of participants including ethnicity. iii) All PNs (maximum 12 if two part-time PNs per practice) will be invited to participate in semi-structured interviews focussing upon their evaluation of the interventions’ acceptability and use in PA consultations. Interviewing with study participants will continue until no new themes are identified (approximately 75 are anticipated, 15 for the ‘non-participants’ and 15 for each of the four groups of trial participants). The qualitative research assistant will also run a focus group at each practice at the end of the trial made up of the practice nurses and the research assistant involved at that practice and a sample of trial participants willing to attend, again reflecting the range of socio-demographic characteristics of participants.

*Participant withdrawal:* Participants will be free to withdraw from the trial at any time and without giving a reason. Practice nurses can advise discontinuation if the intervention poses a hazard to the participant. In both cases, information that has already been collected on participants may still be used and they will be asked if they would be prepared to provide any further data on outcomes at 3 and 12 months (eg questionnaire, anthropometric measurements and / or PA monitoring). Participants who withdraw before they have been randomised will be replaced, those withdrawing or being withdrawn after randomisation will not be replaced.

1. **Study procedures**

*Obtaining informed consent from participants*

The participants will receive a postal invitation to take part in the study accompanied by a patient information sheet summarising the main points of the trial. Those who are interested in participating will return the reply slip, which includes a single, simple screening question about their usual physical activity levels. The research assistant will telephone them to make an appointment, if they are suitable, or will send them a letter to explain why not, if they are unsuitable. The decision regarding participation in the study is entirely voluntary. At the appointment the research assistant will go through the patient information sheet with them and answer any questions that they have about the study. If they are happy to proceed they can sign the study consent form. Patients with dementia, cognitive impairment and learning difficulties will have been excluded from the study by the initial computer search and by the GP checking invitations before they are sent out. However, the research assistant seeking consent will be fully trained in the ethical principles underpinning informed consent and will be able to assess capacity to consent. Participants who have difficulty understanding, speaking or reading English will have been asked to bring a family member or friend with them to the appointment with the research assistant. Copies of the participant information sheet translated into other languages will be available if the practices have indicated that this is appropriate. After obtaining informed consent the research assistant will then continue with the baseline assessment. Patients will have at least a week to reflect further on their participation in the study before they are randomized.

*Randomisation procedure:* Participants completing the baseline assessment (including returning the accelerometer with at least 5 complete days of ≥9 hours / 540 minutes recording) will be allocated to the trial groups by the RA using the Kings College London CTU internet randomisation service to ensure independence of the allocation. Randomisation will be at household level to avoid couple contamination, which could occur if a couple were allocated to different arms. Randomisation will take place after both members of the household have completed baseline assessment. Block randomisation will be used within practice with random sized blocks to ensure balance in the groups and an even workload for nurses. The RA will inform participants by telephone whether they have been allocated to one of the intervention groups or to the control group.

*Baseline, 3 month & 12 month assessment (all participants see flow chart page 14)*

*Baseline assessment*

i)Questionnaire measures – Socio-economic-demographic measures: self-reported ethnic group, self-reported occupation, household composition. Self-reported physical activity: modified Zutphen questionnaire. Health problems: self reported chronic diseases (e.g. heart disease, lung disease, arthritis, depression etc); disability^69^. Patient Reported Outcomes (PROs) (exercise self-efficacy^70^, anxiety and depression (Hospital Anxiety & Depression Scale), perceived health status including mood and pain (EuroQol 5D (EQ-5D)^71^, loneliness^72^. After the participants have worn the accelerometer for 7 days to measure their physical activity levels participants will be asked to complete a short questionnaire about self-reported PA in the last 7 days (General Practice Physical Activity Questionnaire (GPPAQ) International Physical Activity Questionnaire (IPAQ)).

ii)Anthropometric measures – height; weight; waist circumference; hip circumference; body fat, bioimpedence (Tanita monitor);

iii) Objective PA assessment (patient blind to results) measurement of usual physical activity levels (wearing an accelerometer and a blinded pedometer on a belt over one hip, all day for 7 days, only removing for bathing). The monitors and belt will need to be dropped back into the practice after use. The Actigraph (GT3X Manufacturing Technology Inc., Fl. USA) measures vertical accelerations in magnitudes from 0.05-2.0g sampled at 30Hz then summed over a selected (5s) time period, it can record physical activity continuously for up to 21 days. The output, activity counts per unit of time, distinguishes between different walking speeds and physical activity intensities, using standard cut-offs^49;50^. The pedometer function on the accelerometer records steps and will be used for baseline and outcome measurment of step-counts for the trial. The blinded pedometer also records steps and will be used to set an individual step-count target for those randomised to an intervention group, as there can be individual differences in step-counts between the accelerometer and pedometer and participants in the intervention group will be using a pedometer to monitor their own step-counts.

*3 month assessment (postal):* As for baseline assessment, including accelerometer assessment of physical activity, but there is no anthropometric assessment and the questionnaire does not repeat socio-economic-demographic measures, but has additional questions about adverse events, including injuries and health problems over the past 3 months. There are also additional questions for the health economics analysis, assessing the cost to participants.

*12 month assessment (at practice):* As for baseline assessment (including anthropometric assessment and accelerometer assessment) but the questionnaire does not repeat socio-economic-demograhic measures, but has additional questions about adverse events, injuries and health problems over the past 12 months. The control group will additionally be asked about whether they have used a pedometer at any time over the last 12 months.

*Procedure for control group (usual physical activity)*:

After baseline assessment and randomisation, the RA informs participants by telephone that they are in the usual physical activity group and that they should continue as usual with their physical activities throughout the trial. She /he will thank them for participating and inform them that they will be contacted later to arrange the 3 month postal asssessment and the 12 month outcome assessment appointment at the practice, posting out an accelerometer to wear for the 7 days before these. The research assistant will also make contact at 6m & 9m (by telephone, text, email according to patient preference) to check on safety outcomes and contact details. On study completion, the control group will be offered a pedometer, diary and written instructions for a 12 week pedometer-based walking programme either by post or as part of a single consultation with a practice nurse.

*Nature of the complex intervention:* 12 week pedometer-based walking intervention delivered either by post with written instructions or delivered in the context of 3 physical activity consultations with a practice nurse, based on behaviour change techniques.

*Components of the complex intervention*

*i)Pedometers*

Yamax Digi-Walker SW-200 (Tokyo, Japan) is the criterion pedometer with best accuracy^73-75^. It provides direct step-count feedback to participants. Step-counts need daily manual recording and re-setting. The pedometer will be delivered by post along with written instructions on it’s use to the pedometer group, it will be given by the nurse directly to patients in the pedometer plus support group.

ii) *Written instructions and physical activity diaries for a 12-week pedometer based walking intervention (delivered by post to pedometer group, provided in consultations for pedometer plus nurse support group)*

We will have participants’ baseline step-count from their blinded pedometer assessment. They will be given feedback on this and information that adding in 3000 steps/day (approximately equivalent to a 30 minute moderate or brisk walk) on 5 or more days weekly to their baseline would help them to achieve the recommended daily physical activity guidelines. They will also be advised that this can be built up gradually. They will be given advice on the benefits of at least moderate intensity physical activity for health and informed that moderate intensity physical activity makes you feel warm and makes you feel a bit breathless and increases your heart rate, but that you should still be able to talk. A suggested walking plan will be provided as follows:

Weeks 1-2: Add in 1500 steps (approximately a 15 minute moderate or brisk walk) 3 or more days per week

Weeks 3-4: Add in 1500 steps (approximately a 15 minute moderate or brisk walk) 5 or more days per week

Weeks 5-6: Add in 3000 steps (approximately a 30 minute moderate or brisk walk) (or 2 x 15 minute walks) on 3 or more days per week

Weeks 7-8: Add in 3000 steps (approximately a 30 minute moderate or brisk walk) (or 2 x 15 minute walks) on 5 or more days per week

Weeks 9-12: Maintenance, continue adding in 3000 steps (approximately a 30 minute moderate or brisk walk) on 5 or more days per week.

They will be given diaries for recording their daily step-count and walks taken so that they can see if they are acheiving their target for that week. They will be given written advice on how to maintain their activity, and how to anticipate and manage setbacks.

*iii) Physical activity consultations with a practice nurse based on established behaviour change techniques*  A guide to the timing of sessions, session content and Behaviour Change Techniques to be employed is given in Table 1. The techniques used include patient-led goal setting, problem-solving, self-monitoring, ‘SMART’ goals, social support, relapse prevention, addressing barriers, use of written tools including diaries. These techniques have been successfully used by non-specialists in primary care after brief training^76^ and are emphasized in the NHS Health Trainer Handbook^77^ which has been adapted to focus specifically on physical activity behaviour change for use in this trial. Pedometer measurement and diary recording of step-counts provides clear material for physical activity goal-setting, self-monitoring and feedback and should fit very well with this approach. Practice nurse training in these behaviour change techniques to increase PA based on the adapted NHS Health Trainer handbook, combined with training in advising on a 12-week pedometer based walking intervention will be provided.

*Procedure for the pedometer group*

After baseline assessment and randomisation, the RA informs participants by telephone which group they have been allocated to and arranges to post out a pedometer with easy to follow written instructions for a 12 week pedometer-based walking programme. The 12 week programme is based on the participant's own baseline step-count (as assessed by the accelerometer) and advises incremental step-count increases as outlined above. Participants will be given a simple diary to record daily and weekly step-count goals and daily step-counts. Written advice on anticipating and managing setbacks and maintenance following the programme will also be provided. The RA will telephone 1 week after sending out the pedometer to check that it has arrived safely and that it is working properly and to offer a replacement pedometer in the event of loss or malfunction during the 12 week intervention. She will also check that participants understand the 12 week pedometer based walking plan and answer any questions that they have. Arrangements for 3 and 12 month assessments are as for the control group. The RA will also make contact at 6m & 9m (by telephone, text, email according to patient preference) to check on safety outcomes, contact details and progress and to offer support and motivation and a replacement pedometer or batteries, if required. On study completion, participants in this group will be offered a practice nurse PA consultation.

*Procedure for the pedometer plus practice nurse support group*

After baseline assessment and randomisation, the RA informs participants by telephone that they are in this group and arranges an appointment for them with the practice nurse for their first physical activity consultation. The support package consists of a 12 week pedometer-based walking programme with 3 short individually tailored physical activity consultations with a practice nurse (week 1, week 5, week 9). Their baseline step-count (from their blinded pedometer recording) will be reviewed alongside health and anthropometric data, so that an individual physical activity plan, tailored to their baseline step-count abilities, health, goals and personal circumstances and based on increasing walking and walking speed and other existing physical activities, can be produced. At the first consultation the nurse will show participants how to use the pedometer to measure their step-count and how to record step-counts on a weekly diary. The written instructions for the 12 week pedometer-based walking intervention being used by the postal group will also be given to the patients in this group by the nurse, but there can also be individual discussion between the nurse and the patient about what level of step-count increase is most appropriate for them and how fast to increase this. Participants will be asked to wear a pedometer and keep a diary record of daily steps for 4 weeks until they are seen at their next appointment when these will be reviewed with the nurse. If they have achieved their goals, new goals can be set, if not, then problems and barriers and ways of overcoming them can be discussed. Participants can be seen individually or as a couple, for couples both individual goals and opportunities to increase their PA together will be discussed. Arrangements for 3 and 12 month assessments are with the research assistant, as for the control group. The RA will also make contact at 6m & 9m (by telephone, text, email according to patient preference) to check on safety outcomes, contact details and progress and to offer support and motivation and a replacement pedometer or batteries, if required.

*Procedure for qualitative study:*

The qualitative researcher will approach the groups identified in section 5 (participant selection for the qualitative evaluation) and seek their informed consent for a semi-structured telephone interview. He/she will also run a focus group at each practice a the end of the trial, made up of practice nurses and the research assistant involved at that practice and a sample of trial participants. For each group, all interviews will be audio-recorded (unless participants do not consent, when contemporaneous field notes will be taken) and transcribed verbatim professionally. Thematic analysis will proceed in parallel with the interviews to enable refinement of the interview guide and purposive sampling according to emerging themes.

*Procedure for the health economic evaluation*

The economic evaluation will take the perspective of the NHS, personal social services and participants and first undertake a trial based analysis. Participant-level resource use data will be collected for equipment (pedometers), face to face or telephone consultations (length of time and frequency), out of pocket expenses (e.g. transport costs), use of support services (number of calls and contacts by post) and for other health service use (e.g GP attendances, in-patient days, out-patient visits, home visits and services from social services, stays in nursing and residential care). Data will be collected through primary care records, participant questionnaire at 3 and 12 months and monitoring by nurses. Data collection procedures will be merged with effectiveness where possible. Costs that don’t vary by use (e.g. development, production and translation of leaflets) will be costed separately and apportioned to patients within the relevant arm of the trial. Unit costs will be valued using national averages to increase their generalisability.

*End of study definition:* The end of the study for each participant in the nurse support group is when they have attended their 12 month assessment. For the other two groups (control and pedometer plus written instructions) they will be offered a single physical activity consultation with the practice nurse after their 12 month assessment. If they accept this, the end of the study for them will be after this consultation. The end of the study overall is when all patients have completed their 12 month assessments and subsequent consultations with the nurses.

*Participant withdrawal:* If a participant withdraws after signing their informed consent but before completing their baseline assessment (including monitoring their physical activity for 7 days) and before randomisation, they will be replaced with a further participant. Any information already collected on them may still be used.

*Data collection and follow-up for withdrawn subjects:* Participants who withdraw from the trial will be asked if they would be prepared to contribute to further data collection on outcomes at 3 months and 12 months (eg questionnaire, anthropometric measurements and / or physical activity monitoring).

1. **Safety**

*Risks:* The PACE-UP study poses a low risk to patients’ health and to the cause of any serious adverse events. Careful application of the exclusion criteria, from screening primary care records and involving both the GP and patient, will minimize the risk of adverse events. Additionally, the study is based in primary care and patients can discuss concerns with their GP; practice nurses will receive safety training; the intervention is low risk and based on increasing walking and other activities that they already do, not introducing new activities; and the intervention is individualised and starts from the participant’s own baseline activity level with encouragement to start-low-and-go-slow, to minimize risks. However, the information sheets will inform participants and GPs of the potential injury risks, so that consent is obtained with prior knowledge.

*Adverse events:* An adverse event (AE) is any unfavourable and unintended sign, symptom or illness that develops or worsens during the period of observation of the trial. This includes:

1. Exacerbation of a previous illness
2. Increase in frequency or intensity of a pre-existing episodic event or condition.
3. Condition detected after the study started even though it may have been present prior to the study.
4. Continuous persistent disease or symptoms present at baseline that worsen following the study start.

*Serious adverse events:* A serious adverse event (SAE) is any adverse event occurring following study mandated procedures, having received either of the physical activity interventions or usual treatment that results in any of the following outcomes:

1. Death
2. A life-threatening adverse event
3. Inpatient hospitalization or prolongation of existing hospitalization
4. A disability / incapacity

*Causality:* Adverse and serious adverse events can be unrelated, unlikely to be related (non-attributable), possibly or probably related (indirectly attributable) or definitely related to the study (directly attributable). Death from chest infection is a serious non-attributable adverse event and fracture requiring hospitalization from a fall whilst walking is a serious indirectly attributable adverse event. The intervention groups will be asked to increase activities they were already doing, not to take up new ones, so physical activity related adverse events could have happened without them being in the trial, therefore in most cases will be indirectly attributable to the trial.

*Notification and reporting of adverse events:* A standard operating procedure for the management of adverse events will be in place, so that participants or their relatives, practice staff or researchers can inform the chief investigator of any event they consider possibly related to PA promotion. All adverse events reported will be assessed for seriousness, expectedness and causality. All adverse events will be recorded and monitored until resolution, stabilisation or until it has been shown that the intervention is not the cause.

*Notification and reporting of serious adverse events (SAE):* Participants or relatives will be asked to contact the trial site immediately in the event of any SAE. The Chief Investigator shall be informed immediately and shall determine seriousness and causality in conjunction with any treating medical practitioners and another trial physician. SAE need to be reported to the patient’s GP. If the CI determines that the SAE was not causally related to the study intervention then the SAE will be reported to the TSC chair and the sponsor and go on the annual safety report, but no further action is necessary. A serious adverse event that is deemed directly or indirectly attributable to the study intervention and is unexpected (Suspected unexpected serious adverse reaction or SUSAR) will be reported to the Ethics Committee, Trial Steering Committee, R&D department and study sponsor, within 15 days of the CI becoming aware of the event if it is not life-threatening or fatal and within 7 days if it is.

*Retrospective data collection on adverse events:*

*i)Questionnaires:* Intervention and control groups will be sent questionnaires at 3 months and 12 months that will ask specifically about falls, injuries and exacerbation of any pre-existing conditions in the previous 3 month and 12 month periods respectively.

*ii) Contact with research assistant:* The research assistant will contact participants in all 3 groups at 6 and 9 months (by telephone, text or email as preferred by participant) and will ask about adverse events since the last contact.

*ii)Computerised primary care records:* In order to be sure that full data on adverse events is collected, informed consent will be sought to collect data from participants records at the end of the study. All consultation data for the 12 month period of the study for each individual will be downloaded from practice computerised records, including all new problems recorded during this period. Additionally data on all hospital admissions, out of hours attendances and out patient appointments recorded will be downloaded. This will be anonymised before removal from the practice and a researcher who is blind to the intervention or control status of the participants will analyse this data with a standardised proforma recording possible adverse events.

*Monitoring:* Mechanisms will be set up for decision making and governance, with external judgment of the trial’s safety; the Trial Steering Committee (TSC) will assess safety parameters. Precise details on reporting and decision making mechanisms will be agreed at the first TSC meetings, before patient recruitment.

*Annual safety report:*

The chief investigator must report all serious adverse events annually to the REC and the funder.

1. **Statistics**

*Sample size:*

A meta-analysis of a heterogeneous group of short-term intervention studies involving pedometers showed interventions increased steps count per day by 2500 with a SD of 2700^32^. However a smaller increase in steps of 1000 per day would lead to worthwhile health gains if this was sustained for 12 months. We also want to be able to demonstrate whether there are differences in the effects achieved by a pedometer intervention alone compared to a pedometer intervention with nurse support. A sample of 217 patients in each of 3 arms would allow a difference of 1000 steps per day to be detected between any two arms of the trial with a 90% power at the 1% significance level. This means that we will have sufficient power to adjust for multiple hypothesis testing. However, we plan to randomise households. For men and women the effect of clustering is likely to be small but needs to be taken into account when stratifying by age. Assuming an intracluster correlation of 0.5 and an average household size of 1.6 eligible patients we would need to analyse 282 patients per arm. Allowing for approximately 15% attrition we would need to randomise a total of 993 patients (331 control, 331 pedometer only and 331 pedometer plus nurse support). There are 6 general practices (centres) each will recruit approximately 166 patients, approximately 55 to each of the 3 groups.

*Anticipated recruitment:*

We anticipate a conservative recruitment rate of 20% amongst those eligible to participate. This estimate is based on pilot work using pedometers and accelerometers in an observational study of older primary care patients, recruitment rate 43%^49^ and other studies of PA interventions (including with pedometers) amongst middle aged and older adults in primary care, where recruitment has been between 17% and 35%^35;78-81^. Even if our recruitment rate were as low as 10% we would have enough eligible participants (see flow chart page 14)**.**

*Statistical analysis*:

Analysis and reporting will be in line with CONSORT guidelines, with primary analyses being on an intention-to-treat basis, that is, all participants will be included who have outcome data, regardless of their adherence to the interventions. Sensitivity analyses including all randomised patients will be carried out using multiple imputation. All participants will be included in the primary analysis if they have at least 1 satisfactory day of accelerometer recording out of 7 days. A satisfactory recording comprises at least 540 minutes of registered time during a day. Adequacy of the randomisation process to achieve balanced groups will be checked by comparing participant characteristics in the 3 arms (e.g. age, sex, socio-economic group, baseline PA level, health status, body mass index, household size). The same variables will be compared between those who complete follow up and those who drop out completely, and those who fail to provide a complete set of five days data for the primary outcome. Significance tests, either t-test or chi-squared tests, will be used to compare the those with complete data and those who have missing outcomes.

Primary analysis

Estimates of difference between the treatment arms will be based on an analysis of variance model with change in step-count as the dependent variable and allowing for practice and household as random effects. All intervention arms will be compared with each other, adjusting for multiple comparisons. Any variables found to be unbalanced on the baseline analysis will be included in an adjusted analysis. The primary outcome measure is change in step count from baseline to 12 month follow up. Secondary measures which we will also examine are counts per minute, counts per minute of registered time and number of minutes spent in moderate or vigorous physical activity. These measures are likely to be highly correlated with step count and will be analysed using identical approaches to that for step count. The primary analysis will use all patients with outcome data (i.e. complete case analysis, adjusted for covariates likely to predict outcome and missingness). The main outcome will be the average number of steps per day measured over seven days at twelve months. By including baseline number of steps per day as a covariate, this will effectively be measuring change in number of steps over the twelve months. Baseline value of the outcome, practice, season, whether or not taking part as a couple, body mass index, disability and perceived health status will be included as covariates in all analyses. Additionally the patient level covariates will include any variable in the descriptive analysis found to differ markedly between the two intervention arms and any of these variables found to predict missingness. Daily step counts will be analysed using a multi-level random effect regression model allowing for clustering at household level, to compare participants in all the intervention arms. At the day level, day of the week will be added as a covariate to adjust for day of the week, which is particularly important if some days are missing.

Secondary analyses

Subsidiary analysis will investigate whether there is any evidence of interaction, that is whether the treatment effect varies by the following factors: age (<60 versus >=60) gender, socio-economic group, ethnic group, participating as a couple.

A regression model will be used to assess whether change in step-count is related to compliance with the intervention, by including the number of nurse sessions attended (nurse intervention group) and whether or not individual step-count targets were set (nurse intervention group) as variables in the regression model. Numbers in each group who have suffered a fracture, falls and injuries and drop-outs will be compared between the groups using logistic regression in Stata adjusted for clustering.

*Stopping rules:* It would be impossible to carry out interim analyses on sufficient patients to decide to stop, so there are no formal statistical stopping rules. If a patient becomes ineligible, the nurse may discontinue the intervention, but all patients will be asked to complete follow-up assessments. Patients can withdraw at any time.

*Procedure for accounting for missing, unused and unexpected data:* Only days with >540 minutes of registered time on accelerometer on a given day will be used. Multi-level modeling will be used to allow for day of week of measurements. This will allow change within subjects to be estimated, even if less than 7 days of data is provided.

Participants will only be randomized if they provide at least 5 such days of accelerometer data at baseline. Therefore missing baseline data will not be a major problem at baseline but we will use a multilevel linear regression model, taking account of clustering within household and repeat days within individuals to estimate the baseline level for each subject, adjusted for day of the week and days since start of measurement. The potential for missing data at follow-up will also be reduced. The three main covariates, practice, season and whether or not taking part as a couple will be known for all participants, and it is anticipated that most patients will have complete data for other measures. If any covariates have missing values, these will be replaced by the mean value^82^. Multi-level modeling will be used to allow for day of week of measurements. This will allow change within subjects to be estimated, even if less than 7 days of data are provided. The main analysis assumes that, conditional on the model covariates, outcome data are missing at random. This is likely to be true for missing data due to accelerometer failure, and is plausible for missing days and participants who do not return accelerometers. However an alternative plausible assumption is that participants who fail to provide outcome data are less active. Multiple imputation analysis adjusting the imputed values downwards by a range of values will assess the sensitivity of the analysis to the missing at random assumption^82^.

1. **Data handling and record keeping**

*Confidentiality and data protection:*

Only members of the patient's existing clinical care team will have access to records to identify potential participants, check whether they meet the inclusion criteria and make the initial approach to patients. We will ensure that the approach to patients comes from their existing clinical care team. Invitation letter to patients will be sent out by practices from the practice premises. No details on participants will leave the practice until patient consent is given. Participants’ personal data will remain confidential and will be handled, processed and destroyed in accordance with the terms of the Data Protection Act. Dr Tess Harris is the data custodian. Only nurses working in the practice, the research assistant and principal investigator employed by SGUL and authorized research monitors from the Primary Care Trust will have access to non-anonymised data. They are all bound by their organisations ‘policies on data protection.

Personal contact details (e.g. addresses, telephone numbers and emails) and the code linking patient identity with their unique study ID numbers are particularly sensitive information and will not be stored on memory sticks or external hard drives. Paper copies will be kept in a locked filing cabinet in a locked office at the general practices. Electronic copies will be encrypted on a secure encrypted laptop and then transferred to and stored as encrypted files on a secure network drive at SGUL which is saved onto the UNIX system with regular secure back-ups.

All NHS computers, university computers and laptops will be password protected and files will further be further password protected to restrict to those who need access. All laptops and external hard drives used in the study (by the research assistants) will be strongly encrypted by processes at SGUL and files will be password protected. Data collected will be transferred from the laptops onto a secure network drive at SGUL, which is saved on the UNIX system with secure back-ups.

Individuals will be asked specifically about their consent for audio-recording individual qualitative interviews when they consent for the study. The audio-recordings will only be used for research purposes, specifically they will not be used for commercial purposes. Audio-recordings will be stored as digital files (with a unique identifier not a name) and backed up onto the secure SGUL network drive. Arrangements will be made for them to be securely accessed by the professional transcription service

*Maintenance of study documents:* A trial master file with essential documents containing information specific to each phase of the trial (before, during and after) will be kept in a Trial Master File in the Division of Population Health Sciences at St George’s University of London. Site files to reflect the contents of the trial master file will be kept at each practice site.

*Record retention & archiving:* A copy of patient consent forms will be kept at each practice for 12 months after the study has ended before being destroyed. A paper copy of each patient consent form will be transferred to SGUL by the Research Assistant in a secure locked folder when he or she is returning directly to SGUL. Personal data that is identified by patient name or address will be destroyed by 36 months after the study has ended. Other research data from the trial will be stored for 7 years after the study has ended at St George's University of London in the Division of Population Health Sciences & Education. Data will be stored on a secure area network drive, with password protected access restricted to members of the research team. Questionnaires will be archived in a secure locked archiving room. Electronic data will also be archived onto an encrypted external hard drive and stored with the archived questionnaires.

Digital recordings of interviews will be kept for 12 months after the study has ended (stored securely on the network drive at SGUL) and then will be destroyed. They will not be used for commercial purposes.

1. **Ethics, compliance and clinical governance**

*Ethical considerations:* The trial has had successful ethical review by London Research Ethics Committee (Hampstead). The Trial Steering Committee (TSC) will provide overall trial supervision. The main ethical consideration is the risk of harm to participants (Section 7: Safety). Other ethical considerations are the way that recruitment and informed consent are handled, so that potential participants are not put under pressure to take part and the way that confidentiality is maintained (Section 5: Participant Selection, Section 6: Study Procedures & Section 9: Data Handling).

*Compliance:* The trial will be conducted in compliance with the protocol, Good Clinical Practice and regulatory requirements.

*Clinical governance issues:*  National Health Service (NHS) Research & Development (R&D) approval has been given by Primary Care Trusts (PCTs) in the South West London cluster to cover all the practice sites. The research assistants and trial manager will require NHS research passports and letters of access from the PCTs in order to have contact with patients. The PI will also require a letter of access from the PCTs in order to support work in the practices. (If PCTs are replaced with commissioning bodies during the process of the trial, responsibility for governance will be transferred to them).

1. **Monitoring**

*Summary Monitoring Plan*

A clinical trial baseline risk assessment has been carried out and has informed the approach to and extent of monitoring, which is fully documented in the monitoring plan.

*Trial Oversight Committees*

*Trial Management Group (TMG):* TMG members will be TH (chief investigator) SK (statistician) DC (epidemiologist) Sunil Shah (Public health physician) and the trial manager. Meetings will be held monthly, minutes will be retained in the trial master file. The TMG will monitor all aspects of the conduct and progress of the trial, ensure the protocol is adhered to and take action as necessary to safeguard participants and the trial itself. There will be weekly RA supervision meetings by the trial manager, to ensure day-to-day trial management is on track. TH is the project manager with overall responsibility for ensuring timely achievement of milestones. The Pragmatic Clinical Trials Unit will provide advice and support to the TMG on aspects of the trial such as data collection, data storage and data monitoring procedures.

*Trial Investigators’ Group (TIG):*

This group will include all of the trial investigators, they have responsibility for the trial’s conduct and will meet 6 monthly with the research assistants and trial manager to review the progress of the trial. Individual co-investigators are also available between meetings for advice on specific areas as required.

*Trial Steering Committee:* The Trial Steering Committee (TSC) will provide overall trial supervision and ensure that it is being conducted in accordance with Good Clinical Practice. They will monitor the trial progress, including recruitment, data completeness and losses to follow-up and ensure that there are no major deviations from the trial protocol, they will also assess safety parameters. Membership includes an independent chair, Professor Sarah Lewis (statistician, University of Nottingham), an independent member representing the clinical area under study, Professor Paul Little (academic general practitioner, University of Southampton), Mr Bob Laventure, physical activity adviser for the British Heart Foundation to represent the wider public perspective and users views, the Principal Investigator TH and the trial statistician SK. Members of the TSC have been formally appointed by the Health Technology Assessment Programme.The meetings schedule for the TSC and precise details on reporting and decision making mechanisms will be agreed at the first TSC meeting, ahead of patient recruitment. Minutes for TSC meetings will be kept in the trial master file. A Data Monitoring Committee (DMC) will not be required as it would not contribute to the trial, as the risks are low, recruitment and follow-up are over a short period, and it would be impossible to carry out interim analyses on sufficient patients to decide to stop the trial or modify the trial protocol. The TSC chair can convene a DMC if they feel it is required.

*Before the start of recruitment*

TMG, investigator and TSC meetings to review the trial and procedures.

Site visits to review set-up, team understanding of trial procedures etc.

Training of trial manager, research assistants and practice nurses in all aspects of the trial protocol relevant to them.

Input from PCTU on data collection and data monitoring procedures.

*During the trial*

TMG, investigator and TSC meetings to review trial protocol and procedures.

Site visits to review set-up, team understanding of trial procedures, check of essential documents, including signed consent forms etc.

Central data checks.

Recording and reporting of adverse events and serious adverse events (as detailed under section 7).

Input from PCTU on data collection and data monitoring procedures.

PCTU Quality Assurance manager will carry out an audit of the study every 12 months.

Supervision and support for the research assistants will be provided by the trial manager and chief investigator.

Supervision and support will be provided to the nurses from the RAs, trial manager, chief investigator and CBT trainer. The fidelity and quality of the implementation of the intervention will be monitored over time and between different nurses by the following methods: i) analysing the content of a sample of audio-recorded sessions for each nurse by the CBT trainer according to an agreed proforma; ii) completion of a checklist of areas covered in each consultation by the nurse; iii) completion of a nurse patient alliance questionnaire at the end of each patient’s intervention by both the nurse and the patient; iv) analysis of patient step-count diaries from intervention groups returned at 3 month follow-up (photocopied and then returned to patients).

Supervision & quality control for the qualitative RA & evaluation from Professor Victor.

*At the end of the trial*

Check computerized primary care records for adverse events (check accuracy & missing reports).

Site visits to review archiving of trial documents.

*Audit and inspection:*

The trial documentation will be made available to auditors and inspectors representing the sponsor, host institution and regulatory authorities.

Essential documents containing information relating to each phase of the study (before, during and after) will be kept in a trial master file.

1. **Finance and insurance**

*Contracts & Financial Management:* The contract for delivery of the project will be placed with St George’s University of London, they will arrange sub-contracts as appropriate with academic partners.

*Insurance and indemnity arrangements:* SGUL will be the sponsor for the trial as the main employer of the Chief Investigator Dr Harris. The practice nurses will have indemnity arrangements in place with their practices that cover their clinical duties with patients. There are no special compensation arrangements for the trial. If a patient is harmed during the research study there are no special compensation arrangements. If a patient is harmed and this is due to someone’s negligence, they may have grounds for a legal action for compensation against St George’s, University of London, but they may have to pay their legal costs. The normal NHS complaints mechanisms will still be available to them.

1. **Publication and dissemination policy**

Stage 1 (this will occur whatever the results show):

Publish trial protocol and research findings (RCT, health economic evaluation, qualitative evaluation) in high impact factor peer reviewed journals (e.g. Lancet, British Medical Journal etc). Also present prior to publication as posters, parallel & possible plenary sessions at academic conferences (eg Society of Academic Primary Care, Society for Social Medicine etc). Results will also be fed back to participants, practice nurses, practices, the PCTs & other interested parties locally through newsletters & local meetings.

Stage 2 (this will depend upon whether or not both interventions are effective and cost-effective and differences between the interventions in these measures):

If the trial has been well conducted with fidelity of implementation and both interventions were ineffective, then this would be an extremely important negative finding to disseminate to the NHS both locally at practice and PCT level and centrally via NIHR mechanisms.

If the interventions were both effective then differences between them in both effectiveness and cost-effectiveness need to be explored and discussed in terms of effects this could have for policy for the NHS and fed back to the NHS centrally via NIHR mechanisms.

1. **Further follow-up of the PACE-UP trial cohort**

**Rationale:**

PACE-UP analyses at 12 months have shown positive effects on PA levels. We wish to see if this effect is maintained at 3 years, as this has implications for the NHS; specifically, would a future pedometer programme require a “top-up” after 12 months or not.

The primary outcome was change in steps from baseline to 12m. Both intervention groups showed a significant increase in steps/day compared to controls: pedometer by post 641 (95% CI 328,954); pedometer & nurse support 682 (95% CI 371, 994); with no difference between intervention groups 41 (95% CI -272, 354). There was also a significant increase in time in moderate-to-vigorous PA (MVPA) in ≥10 minute bouts (in mins/week) between baseline and 12m compared to controls: postal 33 (95% CI 17, 49); nurse support 35 (95% CI 19,51); intervention group difference 2 (95% CI -14, 18). In a recent paper we estimated that such an increase in PA, if maintained, would reduce coronary heart disease risk by approximately 5% and diabetes risk by 9% (Harris et al PLOS MED 2015); largely independent of change in body weight. The PACE-UP trial has demonstrated that a primary care pedometer based walking intervention can increase objective PA levels in inactive 45-75 year olds, with potential reductions in future cardiovascular risk. Delivery via three nurse PA consultations had the same effect on 12m outcomes as simpler, cheaper postal delivery. This has important implications for delivering PA improvements and lays the ground for a large, pragmatic, postal pedometer intervention delivered through routine primary care. However, it is vital to know whether 12m effects persist or whether a further intervention boost is needed. Little is known about long-term sustainability of PA interventions. A meta-analysis of interventions (including pedometers) to increase PA levels in 55-70 year olds included only 4 trials with data beyond 12m (all self-report). They found a limited evidence base beyond 12m and called for more trials with longer follow-up and objective PA measures (Hobbs et al BMC Med 2013). These findings were supported by a Cochrane systematic review (Hillsdon et al 2013). The recent ProAct 65+ trial (Iliffe et al HTA 2014) found between-group differences persisted at 2 years post-intervention, but this was only for self-reported PA.

We can provide evidence on objective PA levels at 3 years if we extend the PACE-UP follow-up. After the 12m follow-up 212/322(66%) of controls received a pedometer, handbook, diary and an individual step-count target by post. They had no further input (unlike the original postal group, who were telephoned after receiving the pedometer and who returned their diaries for review at 3m). The controls being sent the pedometer by post mimics what would happen if this pragmatic intervention were to be rolled out in routine primary care. Our 3 year follow-up would therefore answer the following: i) Have the original nurse & postal groups maintained increases in objective PA levels seen at 12m? ii) Are there differences between the original nurse & postal groups in objective PA levels at 3 years? (Whilst there were no differences at 12m, at 3m the nurse group had twice the postal group increase. The nurse group may be more motivated to sustain the intervention). iii) Has the simple postal pedometer intervention at 12m increased objective PA levels in this control group (who had previously stable PA levels across all three timepoints)? We will focus on establishing evidence of effectiveness at 3 years in these measures; a cost-effectiveness analysis is not possible, due to group contamination. Anthropometric measures did not show differences at 12 months; our 3 year follow-up will therefore focus on collecting data on the main PA outcomes and other outcomes achievable via postal collection, as this is the most economic way to collect both objective PA accelerometry data and questionnaire data. To allow for seasonal variation in PA levels; baseline, 12m and 3 year outcomes need assessing in the same calendar month, follow-up will therefore run from Oct 2015 to Nov 2016. Our 3 month postal follow-up was excellent (93% 954/1023), demonstrating this is a viable data collection route.

We conducted successful qualitative research by telephone interview in the main trial, (Normansell et al, 2014 BMC Public Hlth). We plan to include a telephone qualitative evaluation in the 3 year follow-up, This would allow exploration of participants’ views about factors encouraging maintenance of change in PA levels and what “top-up” might have helped those whose PA declined by 3 years. The qualitative findings will be invaluable in planning a future, larger, pragmatic postal trial delivered through routine primary care for this age group.

**Postal survey**

*Recruitment and informed consent*

We will approach the original cohort from the trial (n=1023) minus those who withdrew (n=30) to ask them to take part in further follow-up. We will contact the cohort over a 12 month period, from October 2015 to October2016 so that they are able to provide the PA data in the same month that they provided both baseline and 12 month PA data (as season is a powerful determinant of PA levels). Before we contact the participants we will check with their general practices if there have been any deaths or any diagnoses of dementia or terminal illness in the cohort These participants will be excluded. There are no other reasons needed to exclude participants (e.g. other chronic illnesses), as we are not asking participants to increase their PA levels, we just want to monitor what they are currently now doing in their normal routine.

The follow-up will be postal; there will be no requirement to see participants face-to-face. We will post out a letter to them inviting them to take part in the further follow-up study. We will also include results of the trial findings and a participant information sheet explaining about exactly what is involved in the follow-up. A consent form for the follow-up study will also be included. The letter will make it clear that a research assistant from the PACE-UP team will ring them in the week following them receiving the letter, to see if they would like to take part and to answer any questions they might have. If they are happy to participate they would sign the consent form and return the top copy to us in the SAE provided for them. The participant information sheet and the consent form make it clear that participation is voluntary and there will be no effect on their patient care if they choose not to take part, the research assistant will not put pressure on them to participate.

*Data collection*

Once informed consent is obtained, the research assistant will arrange a convenient time to send out the following to participants: a questionnaire about their health and lifestyle; a belt with an accelerometer attached to measure their PA levels for 7 days (identical to one they wore before at baseline, 3 months and 12 months for the original trial); a diary to record their PA for 7 days whilst wearing the belt; and a questionnaire to complete at the end of the 7 days about their PA levels over the previous 7 days. Once they have worn the monitor for 7 days (from getting up in the morning until retiring at night, apart from swimming or showers) they will return the monitor, diary and questionnaires to us in the freepost envelope provided to them. Once we have safely received back the monitor, we will send them a £10 high street gift voucher.

*Outcome measurements*

The outcome measures will be as for the main trial.

Primary outcome: change in average daily step-count, measured over 7 days by accelerometry between baseline and 3 years.

Secondary outcomes: change in time spent in i) at least moderate intensity physical activity (MVPA) and ii) sedentary physical activity between baseline and 3 years, measured over 7 days by accelerometry.

Other outcome measures:

change in self-reported physical activity from the questionnaire (General Practice Physical Activity Questionnaire (GPPAQ) International Physical Activity Questionnaire (IPAQ)

change in other patient reported outcomes from the questionnaire (exercise self-efficacy, anxiety, depression, EQ5D, pain)

*Analysis*

The analyses will follow the same format as for the main trial for primary, secondary and other outcomes given above.

**Qualitative interview study**

We will recruit samples of those in the two original trial intervention groups (pedometer by post and nurse support) selecting some who have maintained their PA increases at 3 years and some who have not (approximately 40 participants to interview in total). Participants will be asked their consent to be approached for a telephone interview on the follow-up study consent form. Verbal consent will also be obtained by the research assistant prior to audio-recording the interview. Participants will be asked their views about factors encouraging maintenance of change in PA levels and what “top-up” might have helped those whose PA declined by 3 year follow-up. A sample of control participants who received the pedometer postal intervention at 12 months will also be approached for telephone interview; some who subsequently increased their PA levels and some who did not (approximately 20 in total). They will be asked their views about the success or otherwise of the minimalist postal pedometer intervention. Those taking part in the interview will be offered a £10 gift voucher after completion of the interview.

The audio-recordings from the interviews will be professionally transcribed and then thematic analysis will be conducted on the transcripts. Christina Victor (qualitative trial co-investigator) will supervise the analysis and writing up of the qualitative interview data.

1. **PACE-UP trial implementation work – “PACE-UP Next Steps”**

**Rationale for implementing PACE-UP**

*Findings from main trial*

The PACE-UP trial recruited 10% of those invited (1023/10,467). 12 month findings showed significant effects of both interventions on the primary outcome, steps/day and the main secondary outcome, weekly time spent in moderate-to-vigorous physical acitivty (MVPA) in bouts. Additional steps/day compared to the control group were 677 for nurse support (95% C.I. 365-989) and 642 for postal (95% C.I. 329-955). Additional MVPA in bouts (minutes/week) were 35 for nurse support (95% C.I. 19-51) and 33 for postal (95% C.I. 17-49). There were no significant differences between the two intervention groups at 12 months. The intervention was safe, there was no increase in adverse events in the intervention groups compared to control, whether for self-reported questionnaire adverse events, spontaneously reported serious adverse events or adverse events from computerised GP records for those giving their written consent for download of this data (1005/1023, 98%). There was also a suggestion from GP data that cardiovascular events were significantly lower at 12 month follow-up in the intervention group than the control group (p=0.04) (although this was based on small numbers of events). These findings have been published in full.^83^ Health economic analyses also demonstrated that the postal intervention provided cost-effective long-term quality-of-life benefits^84^.

*Findings from 3-year follow-up*

We followed up 67% of the original trial cohort (681/1023) at 3 years. Findings at 3 years showed persistent intervention effects, with again no statisically significant difference between them. Additional steps/day compared to the control group were 670 for nurse support (95% C.I. 237-1102) and 627 for postal (95% C.I. 198, 1056). Additional MVPA in bouts (mins/week) were 24 for nurse support (95% C.I. 3, 45) and 28 for postal (95% C.I. 7-49).

*The case for implementation*

These findings taken together suggest that a pedometer-based intervention, delivered either by post or with minimal support can bring about significant increases in both step-counts and time in MVPA in a cost-effective manner, with persistent effects still present at 3-year follow-up. From other literature we know that sustained increases in step-counts and MVPA of these sizes can lead to significant health effects, in our main trial outcomes paper we estimated the approximate reductions in risk that could be achieved eg reductions in heart disease of approximately 4.5% (95% C.I. 3-6%) and all-cause mortality of approximately 4% (95% C.I. 1-7%)^83^.

We therefore believe that we have a cost-effective intervention that could be rolled out in routine primary care, which could make an important long-term contribution to addressing the public health physical activity (PA) challenge. We intend to seek funding for a phase IV evaluation from the National Institute of Health Research to establish the place of this intervention in routine NHS primary care. There are however three important outstanding questions that need to be addressed before we undertake this large scale evaluation.

1. *Recruitment.* Recruiting randomly selected participants aged 45-75 years through the post gave an uptake of only 10% overall and lower in invitees from socio-economically deprived groups and South Asian ethnic groups. Could we increase the reach of our intervention, particularly for these hard to reach groups, by recruiting through primary care consultations with a GP or nurse for chronic health conditions where a PA intervention is indicated (for example diabetes, heart disease, stroke, hypertension, chronic obstructive pulmonary disease, arthritis)? Could we also increase recruitment by offering the intervention as part of an NHS Health Check, where low PA levels have been identified?
2. *New Technology.* Since the trial was undertaken there has been a dramatic increase in the use of wearable technology for measuring personal PA levels (eg smartphones, fitbits, garmins, etc) and online monitoring. Further research into how the PACE-UP 12-week PA programme could be integrated into use of these devices (with or without a pedometer) is needed.
3. *Robust outcome evaluation using data from primary care records.* It is not clear how we would best measure the outcome of a large phase IV evaluation. It is difficult to measure PA objectively on very large numbers of people (the accelerometry used in our trial for approximately 1000 people would be too difficult and expensive to carry out for a large scale phase IV evaluation delivered through routine care). Self-report questionnaire PA measures (e.g GPPAQ the General Practice Physical Activity Questionnaire, that is sometimes used in primary care) are prone to recall bias and not valid as a primary outcome measure for an evaluation study. We had a signal from the primary care data downloaded at 12 months on those giving written permission for this (1005/1023, 98% of the cohort) that cardiovascular events were lower in the intervention groups than in the control group (p=0.04), but this was based on small numbers of events (2 in the nurse group, 1 in the postal group and 8 in the control group). Further download of GP data at 3-4 years on the same initial trial cohort would give us a larger number of accumulated events and would inform us whether monitoring of cardiovascular events from routine primary care data could give us a robust evaluation outcome measure.

Before we apply for funding for a large-scale evaluation, we need to conduct a feasibility study of PACE-UP implementation to address these outstanding questions.

**PACE-UP next steps implementation study**

We have obtained funding for this feasibility work from two sources. Firstly, we have been adopted by the CLAHRC (Collaboration for Leadership in Applied Health Research & Care) South London and awarded £20k funding. Secondly, we have been awarded £30k by St George’s University of London, Strategic Development Award. The protocol for this feasibility work has been peer reviewed by these funders.

1. *Recruitment*

We plan to test out three different methods of recruiting patients to the intervention using three of the South London practices that were involved in the original PACE-UP trial. The methods are as follows:

1. Face-to-face consultation recruitment of 45-75 year olds who are seeing either the GP or the practice nurse

for a chronic health condition, where the practitioner has assessed that PA levels are low and that the patient could benefit from a walking intervention. The health conditions can be any that the practitioner feels would benefit and include (but are not limited to) the following: type 2 diabetes; ischaemic heart disease; stroke or transient ischaemic attack; hypertension, chronic obstructive pulmonary disease, arthritis, back pain;

1. Face-to-face NHS health check recruitment of patients aged 45-75 years having NHS Health Checks who are

identified by the nurse or health care assistant as having low PA levels, who might benefit from a walking intervention;

1. Postal NHS health check recruitment of 45-75 year old patients who have had an NHS Health Check in the

last 12 months and were recorded on the GP system as having low PA levels, who might benefit from a walking intervention.

We aim to recruit approximately 40 patients from each of these routes, ie approximately 120 patients in total. We will count the number of patients who are offered the intervention in each recruitment method, so that we can estimate the recruitment rate from each approach.

In both face-to-face methods the practice nurse / GP or health care assistant will introduce the study to the patient during their consultations and if they are interested they will hand them a pack including the appropriate patient information sheet and consent form (a slightly different information sheet and consent form for each recruitment route). The pack will also contain the PACE-UP patient handbook and PA diary, exactly as were used in the PACE-UP trial and details about online PACE-UP support and a mobile app (see new technology section below), that can be used either alongside or instead of the paper diary to record their PA levels, according to their preference. There will be a freepost reply envelope to return the consent form to the researchers at St George’s University of London, if they wish to participate.

For the group recruited by post following a prior NHS Health Check, a list of eligible patients will be created at the practice by practice administrative staff searching on the practice computer system for those who have had an NHS Health Check in the last 12 months who were found to have low PA levels from the online questionnaire used as part of the NHS health check. A patient letter will then be posted out from the practice, inviting them to participate, along with the appropriate patient information sheet and consent form. There will be a freepost reply envelope to return the consent form to St George’s University of London (SGUL), if they wish to participate. Once this is received the research assistant will post out a pack containing a pedometer, the PACE-UP patient handbook and PA diary, exactly as were used in the trial and also details about online PACE-UP support and a mobile app (see new technology section below), that can be used either alongside or instead of the paper diary to record their PA levels, according to their preference.

For all groups, the patient information sheets clearly describe what taking part would involve for participants. They state that PACE-UP has been shown to be a safe and effective primary care walking intervention in their age group. They will be encouraged to consult their GP as usual if they develop any new health problems. They are advised not to take part if they have had recent falls, poor vision or balance, or if they have had a heart attack or heart operation in the last 3 months. If they are unsure whether it is safe for them to increase their walking, they are encouraged to discuss this with their GP.

The research assistant at SGUL will be available by telephone or email if participants have any queries about the study. Participants recruited via all three methods will be encouraged to send back the completed patient diary to SGUL researchers after the 12-week intervention, if they have opted to record their PA in the diary.

The consent forms include a request for the research assistant to conduct a short telephone interview with the participant on their experience of being recruited into the study, their thoughts on the paper diary and handbook, as well as positive and negative feedback on the online resources and the mobile app. Participants who provide consent for this part of the study will be contacted to arrange a short telephone interview. The interview will be audio-recorded and verbal consent for this will be sought before commencing the interview.

The consent forms also include a request to access the participant’s GP records. This is so that we can get information on their medical history and current medications, without burdening them with a questionnaire for this information. No consultation text will be accessed.

1. *New Technology – development and testing of PACE-UP online resources and mobile app*

*Development*

We are developing an online version of the PACE-UP paper-based intervention (handbook and diary) which can be used instead of or alongside the paper-based version and will be offered as part of the pack including the pedometer, handbook and diary to all participants, regardless of recruitment route. We are collaborating with the eLearning unit at St George’s University of London, which has extensive app development and testing expertise and developing a PACE-UP mobile app to accompany the website, to be used with either android or apple devices. The app will be free for participants, distributed through the app stores on each platform and will provide the opportunity for participants to enter or review their data on-the-go. The website developer and the app developer will work closely together and carry out joint initial testing, to ensure that the online resource is developed in an appropriate way for the app to be built successfully onto it and that both synergise with paper resources.

*Testing and evaluation*

The research assistant will follow-up all the participants. Their engagement with the online resources and mobile app will be apparent from the step-count and other PA data that they have recorded directly through the website or app, including how often they entered data, how many weeks they recorded for and whether or not they met their individual step-count targets. For those who preferred to use written materials, we will have asked them to return their completed step-count diaries after the 12-week intervention and the levels of completion can be assessed. Qualitative interviews will be carried out with a sample of participants recruited from each of the different routes choosing to use either online, mobile app, written materials or a combination, who give consent to be interviewed. The interviews will explore how easy the different resources were to use, any problems encountered, any suggestions for improvements and whether they intend to continue monitoring their PA levels and if so, which tools they would prefer to use to help them to do this.

1. *Robust outcome evaluation using routine primary care data for original trial participants at 3-year follow-up*

We plan to download routine data from participants’ primary care records for those who gave us written consent to access their records at the start of the trial (see trial consent form approved by ethics, version 1.0 dated 15^th^ March 2012). The searches used to download data at 12 months would be run again in each of the seven original trial practices for those 98% (1005/1023) giving consent, who are still registered at the practices, minus those who have subsequently withdrawn from the trial (n=216). The searches will download the following information: number and date of consultations and Read codes for diseases (including those arising from hospital admissions) for an approximate 3-4 year period following date of informed consent. This will allow analyses of different outcomes including new onset cardiovascular events (myocardial infarction, coronary artery bypass graft, angioplasty, ischaemic heart disease, angina, transient ischaemic attack and stroke), fractures and injurious falls. The proportions developing new cardiovascular events, fractures or falls in the three year follow-up period will be compared between the three groups using Chi-Squared tests or Fisher exact tests. No text data from consultations will be downloaded. Data will be anonymised before removing from the practice (no name, address, or NHS number) the only identifier will be the study ID code. Data will be stored securely on an SGUL encrypted laptop in password protected files to transfer it from the practice to SGUL, where it will then be transferred onto a secure, encrypted drive on the SGUL server.

**References**

1. **Appendices**

*Table 1**: Behaviour change techniques used in physical activity consultations with the practice nurse Table 2: Behaviour change techniques included in the PACE-UP trial Handbook and diary*

**Table 1: Details of the behaviour change techniques to be used in physical activity consultations with the practice nurse in the PACE-UP trial**

| **Week** | **Sessions** | **Guide to session content** | **Behavioural Change Techniques used (Michie et al 2011^66^)** |
| --- | --- | --- | --- |
| 1 | Session1:  First steps (30 minutes)  Week1 | Review health status, current activity, health benefits of physical activity  Cost-benefit analysis for increasing physical activity  Physical activity guidelines & how to increase PA safely  Moderate intensity physical activity and relating it to number of steps  Review participant baseline step-count  Teach use of pedometer & recording walks and steps in diary  Ideas for increasing steps  Goal-setting – PACE-UP goals or tailored to the individual patient  Use of rewards for effort and for achieving goals  Summarise and check patient understanding, plan time for for next meeting  Communication strategies to overcome resistance and promote patient-led change | 1,2,  2,  4,21,  19,  21, 26,  20,  7,9,16,  12,13,  37 |
| 5 | Session 2:  Continuing the changes (20 minutes)  Week 5 | Review step-count and walking diary  Encourage progress in increasing walking and achieving step-count goals  Troubleshoot any problems with pedometer or diary  Review target and agree goals for next stage  Barriers and facilitators to increasing physical activity, overcoming barriers, encouraging support  Pacing and avoiding boom and bust  Check confidence levels, build confidence to make change  Summarise & check patient understanding, plan time for next meeting  Communication strategies to overcome resistance and promote patient-led change | 10,19,  12,13,  8  7,9,16,  8,  29,  9, 35  5, 18, 29, 36  37 |
| 9 | Session 3:  Building lasting habits (20 minutes)  Week 9 | Review step-count and walking diary  Review overall progress over the sessions  Encourage progress in increasing walking and achieving goals  Preparing for setbacks  Building habits: discuss methods of maintaining lasting change, including repetition, if-then rules and support  Setting goals: maintaining current activity or increasing further?  Remind re contact with research assistant in 3-4 weeks  Communication strategies to overcome resistance and promote patient-led change | 10,19,  11,  12,13,  35,  7, 29,23,35,  7,9,16,26    37 |

1. Provide general information on behaviour-health link; 2. Provide information on consequences to individual; 4. Provide normative information about others’ behaviour; 7. Action planning; 8. Barrier identification; 9. Set graded tasks; 10. Prompt review of behavioural goals; 11. Prompt review of outcome goals; 12. Prompt rewards contingent on effort; 13. Prompt rewards contingent on successful behaviour; 16. Prompt self-monitoring of behaviour; 18. Prompting focus on past success; 19. Provide feedback on performance; 20. Provide information on when and where to perform the behaviour; 21. Provide instructions on how to perform the behaviour; 26. Prompt practice; 29. Plan social support / social change; 35. Relapse prevention / coping planning; 36.Stress management / emotional control training; 37. Motivational interviewing.

Table 2: PACE-UP patient handbook and diary and behavioural change techniques included

|  | **Guide to content** | **Behavioural Change Techniques (Michie et al 2011^66^)** |
| --- | --- | --- |
| **Patient handbook** | Health benefits of increasing walking  Physical activity guidelines  Moderate intensity physical activity and relating it to number of steps  PACE-UP walking programme and step-count targets  Review participant baseline step-count  How to increase PA safely  Useful websites  How to keep going when PACE-UP programme finishes | 1,2,  4,  7,9,16,  19,  21,  4  16, 1,2, 26, 29, 35 |
| **Patient diary** | How to use pedometer and record steps in diary  Frequently asked questions on PACE-UP trial  Weekly recording of step-count and walking in diary (weeks 1-12)  Achievement of targets (weeks 1-12)  Planning when to walk, where to walk, who to walk with  Week 2 tips and motivators: make walking part of your daily routine  Week3 tips and motivators: remember personal benefits, what to do if you are falling behind your targets  Week 4 Keep it up: praise and reward yourself, encouraging social support  Week 5 Keep motivated: write down step-counts, ask for support  Week 6 Now we are moving: obstacles and solutions  Week 7 How to make these changes permanent – ideas for new walks, making time for walking, what gains have been made so far?  Week 8 Maintain the gain: pacing, tips for safe exercising  Week 9 Be busy being active: keep monitoring with pedometer, places, people and thoughts that motivate you  Week 10 Change does not happen in a straight line! Preparing for setbacks  Week 11 Make it a healthy habit: building regular exercise habits, creating if-then plans  Week 12 I’ve changed: how to keep up your walking programme  Congratulations you have completed the programme  How to keep going when PACE-UP programme finishes | 16, 21  7,9,19,26  10,12,13,  20,29,  20,  2, 20, 35  35,  12,13,29,  12, 16, 29  8,  38,17, 11  9,21, 35  16, 24,29,36,  35,  1,2,7, 23,  16, 20, 29,  11, 16,17,  1, 16, 29 |

1. Provide general information on behaviour-health link; 2. Provide information on consequences to individual; 4. Provide normative information about others’ behaviour; 7. Action planning; 8. Barrier identification; 9. Set graded tasks; 10. Prompt review of behavioural goals; 11. Prompt review of outcome goals; 12. Prompt rewards contingent on effort; 13. Prompt rewards contingent on successful behaviour; 16. Prompt self-monitoring of behaviour; 17. Prompting self-monitoring of behavioural outcome; 19. Provide feedback on performance; 20. Provide information on when and where to perform the behaviour; 21. Provide instructions on how to perform the behaviour; 26. Prompt practice; 29. Plan social support / social change; 35. Relapse prevention / coping planning; 36.Stress management / emotional control training.
