# Supplementary material for: Effect of pedometer-based walking interventions on long-term health outcomes: Prospective 4-year follow-up of two randomised controlled trials using routine primary care data
Source: PLoS Med. 2019 Jun 25;16(6):e1002836. doi: 10.1371/journal.pmed.1002836 (PMC6592516; doi:10.1371/journal.pmed.1002836)
Supplement: S2 Text — (DOCX) [file pmed.1002836.s003.docx]

**S2_Text: CONSORT 2010 checklist of information for PACE-UP randomised controlled trial 4-year primary care outcomes paper**

Details relating to the PACE-UP trial are reported in the main trial outcome paper [1] and trial protocol paper [2]. Details provided here for clarity.

| Section/Topic | Item No | Standard Checklist item | Where information is provided in 4-year primary care outcomes paper or other sources |
| --- | --- | --- | --- |
| Title & Abstract | 1a | Identification as a randomised trial in the title YES | Title of 4-year primary care outcomes paper. |
|  | 1b | Structured summary of trial design, methods, results, & conclusions (see CONSORT checklist for abstracts table 2 below). YES | Abstract (full details included in main trial outcomes paper [1]) See also table 2 below. |
| Background and objectives | 2a | Scientific background and explanation of rationale YES | Introduction of 4-year primary care outcomes paper. |
|  | 2b | Specific objectives or hypotheses YES | Introduction of 4-year primary care outcomes paper. |
| Trial design | 3a | Description of trial design (such as parallel, factorial) including allocation ratio. YES | Details in main trial outcome paper [1] Methods, study design. |
|  | 3b | Important changes to methods after trial commencement (such as eligibility criteria), with reasons YES | Details in main trial outcome paper [1] Methods, statistical analyses. |
| Participants | 4a | Eligibility criteria for participants  YES | Details in main trial outcome paper [1] Methods, study design participants and trial protocol paper [2]. |
|  | 4b | Settings and locations where the data were collected. YES | Details in main trial outcome paper [1] Methods, study design & participants. |
| Interventions | 5 | The interventions for each group with sufficient details to allow replication, including how and when they were actually administered. YES | Details in main trial outcome paper [1] & trial protocol paper [2]. Also summarised Table 1 of 4-year primary care outcomes paper. |
| Outcomes | 6a | Completely defined pre-specified primary and secondary outcome measures, including how and when they were assessed YES | Methods, procedures and outcome measures of 4-year primary care outcomes paper and protocol for extended follow-up (Supplementary Text 1). |
|  | 6b | Any changes to trial outcomes after the trial commenced, with reasons | Not applicable. |
| Sample size | 7a | How sample size was determined YES | Details in main trial outcome paper [1] Methods, statistical analysis and trial protocol paper [2] Methods, sample size.  Also Methods, sample size of 4-year primary care paper. |
|  | 7b | When applicable, explanation of any interim analyses and stopping guidelines | Not applicable |
| Sequence generation | 8a | Method used to generate the random allocation sequence YES | Details in main trial outcome paper [1] Methods, randomisation and masking. |
|  | 8b | Type of randomisation; details of any restriction (such as blocking and block size) YES | Details in main trial outcome paper [1] Methods, randomisation and masking. |
| Allocation concealment mechanism | 9 | Mechanism used to implement the random allocation sequence (such as sequentially numbered containers), describing any steps taken to conceal the sequence until interventions were assigned. YES | Details in main trial outcome paper [1] Methods, randomisation and masking. |
| Implementation | 10 | Who generated the random allocation sequence, who enrolled participants, and who assigned participants to interventions. YES | Details in main trial outcome paper [1] Methods, randomisation and masking. |
| Blinding | 11a | If done, who was blinded after assignment to interventions (for example, participants, care providers, those assessing outcomes) and how. YES | Details in main trial outcome paper [1] Methods, randomisation and masking. |
|  | 11b | If relevant, description of the similarity of interventions. YES | Details in main trial outcome paper [1]. Also summarised Table 1 in 4-year primary care outcomes paper. |
| Statistical methods | 12a | Statistical methods used to compare groups for primary and secondary outcomes YES | Methods, statistical analysis in 4-year primary care outcomes paper. |
|  | 12b | Methods for additional analyses, such as subgroup analyses and adjusted analyses. YES | Methods, statistical analysis in 4-year primary care outcomes paper. |
| Participant flow (a diagram is strongly recommended) | 13a | For each group, the numbers of participants who were randomly assigned, received intended treatment, and were analysed for the primary outcome YES | Figure 1a (CONSORT diagram for 4-year primary care outcomes paper). |
|  | 13b | For each group, losses and exclusions after randomisation, together with reasons YES | Figure 1a (CONSORT diagram for 4-year primary care outcomes paper). |
| Recruitment | 14a | Dates defining the periods of recruitment and follow-up YES | For main trial see trial outcome paper [1] Methods, study design and participants. For 4-year primary care data see Methods, study design and participants. |
|  | 14b | Why the trial ended or was stopped | Not applicable |
| Baseline data | 15 | A table showing baseline demographic and clinical characteristics for each group YES | Table 2 in 4-year primary care outcomes paper. |
| Numbers analysed | 16 | For each group, number of participants (denominator) included in each analysis and whether the analysis was by original assigned groups YES | CONSORT diagram Figure 1a in 4-year primary care outcomes paper. |
| Outcomes and estimation | 17a | For each primary and secondary outcome, results for each group, and the estimated effect size and its precision (such as 95% confidence interval). YES | Results, Table 3 and Table 4 in 4-year primary care outcomes paper. |
|  | 17b | For binary outcomes, presentation of both absolute and relative effect sizes is recommended | For relative effect sizes, Table 3 and Table 4 in 4-year primary care outcomes paper. For absolute effect sizes and NNT Table 5 in 4-year primary care outcomes paper. |
| Ancillary analyses | 18 | Results of any other analyses performed, including subgroup analyses and adjusted analyses, distinguishing pre-specified from exploratory. YES | NA |
| Harms | 19 | All important harms or unintended effects in each group (for specific guidance see CONSORT for harms) YES | See main trial outcome paper [1] Results, Table 4. |
| Limitations | 20 | Trial limitations, addressing sources of potential bias, imprecision, and, if relevant, multiplicity of analyses.  YES | Details in main trial outcome paper [1] Discussion, study strengths and limitations. Details relating to 4-year primary care data outcomes in Discussion (paragraph on strengths and weaknesses of the study). |
| Generalisability | 21 | Generalisability (external validity, applicability) of the trial findings. YES | Details in main trial outcome paper [1]. Relating to 4-year primary care outcomes, Discussion, (paragraph on strengths and weaknesses of the study). |
| Interpretation | 22 | Interpretation consistent with results, balancing benefits and harms, and considering other relevant evidence. YES | Relating to 4-year primary care outcome data, Discussion, (paragraph comparison with other studies and paragraph implications for clinicians, researchers and policy makers). |
| Registration | 23 | Registration number and name of trial registry YES | At end of abstract. |
| Protocol | 24 | Where the full trial protocol can be accessed, if available  YES | Published trial protocol paper [2]. Open access online journal & available on PACE-UP website [www.paceup.sgul.ac.uk](http://www.paceup.sgul.ac.uk) For 4-year primary care outcomes, protocol approved by ethics available as Supplementary Text 1. |
| Funding | 25 | Sources of funding and other support (such as supply of drugs), role of funders YES | Included in PLoS Med online funding declaration submission |

**Table 2: CONSORT checklist for abstracts**

| Item | Standard Checklist item | Included in main trial outcomes paper abstract [1] |
| --- | --- | --- |
| Title | Identification of study as randomised. | YES |
| Trial design | Description of the trial design (e.g. parallel, cluster, non-inferiority) | YES |
| Methods |  |  |
| Participants | Eligibility criteria for participants and the settings where the data were collected. | YES |
| Interventions | Interventions intended for each group. | YES |
| Objective | Specific objective or hypothesis. | YES |
| Outcome | Clearly defined primary outcome for this report. | YES |
| Randomization | How participants were allocated to interventions. | YES |
| Blinding (masking) | Whether or not participants, care givers, and those assessing the outcomes were blinded to group assignment. | YES |
| Results |  |  |
| Numbers randomized | Number of participants randomized to each group. | YES |
| Numbers analysed | Number of participants analysed in each group. | YES |
| Outcome | For the primary outcome, a result for each group and the estimated effect size and its precision. | YES |
| Harms | Important adverse events or side effects  This was not a primary or secondary  outcome, but they are reported in paper. | NO |
| Conclusions | General interpretation of the results. | YES |
| Trial registration | Registration number and name of trial register. | YES |
| Funding | Source of funding. | YES |

**References:**

1. **Harris T**, Kerry SM, Limb ES, Victor CR, Iliffe S, Ussher M, et al. Effect of a Primary Care Walking Intervention with and without Nurse Support on Physical Activity Levels in 45- to 75-Year-Olds: The Pedometer And Consultation Evaluation (PACE-UP) Cluster Randomised Clinical Trial. ***PLoS Med.*** 2017;14(1):e1002210. doi: 10.1371/journal.pmed.1002210. PubMed PMID: 28045890; PubMed Central PMCID: PMCPMC5207642.

2. **Harris T**, Kerry SM, Victor CR, Shah SM, Iliffe S, Ussher M, et al. PACE-UP (Pedometer and consultation evaluation - UP) - a pedometer-based walking intervention with and without practice nurse support in primary care patients aged 45-75 years: study protocol for a randomised controlled trial. ***Trials.*** 2013;14:418. doi: 1745-6215-14-418 [pii];10.1186/1745-6215-14-418 [doi].
