# Supplementary material for: Effect of pedometer-based walking interventions on long-term health outcomes: Prospective 4-year follow-up of two randomised controlled trials using routine primary care data
Source: PLoS Med. 2019 Jun 25;16(6):e1002836. doi: 10.1371/journal.pmed.1002836 (PMC6592516; doi:10.1371/journal.pmed.1002836)
Supplement: S5 Text — (DOCX) [file pmed.1002836.s006.docx]

**S5_Text: Comparison of physical activity levels from our study with cohort studies for estimating cardiovascular and type 2 diabetes relative risks**

For CVD and T2DM incidence we have extracted data from Table 4 of Wahid et al which present estimates of the relative risk of an increase of 11.25 MET h/week of physical activity – this equates to 150 minutes of MVPA/week. We have used the Log Linear estimates (final column) to simplify the calculation.

In order to compare the estimates from Wahid et al to the estimates from our trials it is necessary to allow for the fact that our interventions only increased MVPA by 30 minutes per week.

Under the log-linear assumption the relative risks from Wahid et al need to be raised to the power .2 (=1/5) to reflect the fact that in our trial we only increased MVPA on average by 30 minutes compared to the 150 minutes in Wahid et al.

**Outcome RR (95%CI) for 150 mins MVPA/wk RR (95%CI) for 30 mins MVPA/wk**

CVD incidence 0.91 (0.85, 0.97) 0.98 (0.97, 0.99)

T2DM incidence 0.69 (0.67, 0.71) 0.93 (0.92, 0.93)
