# Supplementary material for: Effect of pedometer-based walking interventions on long-term health outcomes: Prospective 4-year follow-up of two randomised controlled trials using routine primary care data
Source: PLoS Med. 2019 Jun 25;16(6):e1002836. doi: 10.1371/journal.pmed.1002836 (PMC6592516; doi:10.1371/journal.pmed.1002836)
Supplement: S1 Table — (DOCX) [file pmed.1002836.s007.docx]

|  |  | |  | **Unadjusted** | | | **Adjusted for study, sex, age** | | |
| --- | --- | --- | --- | --- | --- | --- | --- | --- | --- |
|  | | **Cox regression models** | | **Haz ratio** | **(95% CI)** | ***p*-value** | **Haz ratio** | **(95% CI)** | ***p*-value** |
|  | | **Non-fatal cardiovascular events** | | |  |  |  |  |  |
| Group |  | | Intervention vs Control | 0.24 | (0.07, 0.75) | 0.01 | 0.24 | (0.07, 0.77) | 0.02 |
| Study |  | | PACE-UP vs PACE-Lift |  |  |  | 0.80 | (0.24, 2.64) | 0.71 |
| Sex |  | | Male vs Female |  |  |  | 0.61 | (0.19, 1.94) | 0.40 |
| Age |  | | per year |  |  |  | 1.11 | (1.02, 1.20) | 0.01 |
|  |  | |  |  |  |  |  |  |  |
|  | | **Non-fatal and fatal cardiovascular events** | | | |  |  |  |  |
| Group |  | | Intervention vs Control | 0.32 | (0.12, 0.87) | 0.03 | 0.34 | (0.12, 0.91) | 0.03 |
| Study |  | | PACE-UP vs PACE-Lift |  |  |  | 1.07 | (0.38, 3.02) | 0.89 |
| Sex |  | | Male vs Female |  |  |  | 1.03 | (0.39, 2.71) | 0.96 |
| Age |  | | per year |  |  |  | 1.11 | (1.03, 1.19) | 0.01 |
|  | |  | | | | | | | |
|  | | **Diabetes diagnosis** | |  |  |  |  |  |  |
| Group |  | | Intervention vs Control | 0.76 | (0.42, 1.36) | 0.35 | 0.75 | (0.42, 1.36) | 0.34 |
| Study |  | | PACE-UP vs PACE-Lift |  |  |  | 0.74 | (0.34, 1.61) | 0.44 |
| Sex |  | | Male vs Female |  |  |  | 0.99 | (0.54, 1.82) | 0.99 |
| Age |  | | per year |  |  |  | 1.03 | (0.99, 1.07) | 0.14 |
|  |  | |  |  |  |  |  |  |  |
|  | | **Depression diagnosis** | |  |  |  |  |  |  |
| Group |  | | Intervention vs Control | 1.04 | (0.49, 2.18) | 0.92 | 0.98 | (0.46, 2.07) | 0.96 |
| Study |  | | PACE-UP vs PACE-Lift |  |  |  | 0.69 | (0.22, 2.15) | 0.52 |
| Sex |  | | Male vs Female |  |  |  | 1.13 | (0.54, 2.35) | 0.75 |
| Age |  | | per year |  |  |  | 0.98 | (0.94, 1.03) | 0.49 |
|  |  | |  |  |  |  |  |  |  |
|  | | **Fractures** | |  |  |  |  |  |  |
| Group |  | | Intervention vs Control | 0.55 | (0.35, 0.88) | 0.01 | 0.56 | (0.35, 0.90) | 0.02 |
| Study |  | | PACE-UP vs PACE-Lift |  |  |  | 0.91 | (0.50, 1.66) | 0.77 |
| Sex |  | | Male vs Female |  |  |  | 0.63 | (0.37, 1.05) | 0.08 |
| Age |  | | per year |  |  |  | 1.03 | (1.00, 1.07) | 0.06 |
|  |  | |  |  |  |  |  |  |  |
|  | | **Negative binomial models** | | **Unadjusted** | | | **Adjusted for study, sex, age** | | |
| **Falls** |  | |  | **IRR** | **(95% CI)** | ***p*-value** | **IRR** | **(95% CI)** | ***p*-value** |
| Group |  | | Intervention vs Control | 1.05 | (0.76, 1.43) | 0.78 | 1.07 | (0.78, 1.46) | 0.67 |
| Study |  | | PACE-UP vs PACE-Lift |  |  |  | 1.07 | (0.73, 1.57) | 0.71 |
| Sex |  | | Male vs Female |  |  |  | 0.62 | (0.45, 0.86) | 0.004 |
| Age |  | | per year |  |  |  | 1.03 | (1.01, 1.05) | 0.005 |
|  |  | |  | **α** | **(95% CI)** | ***p*-value for LR test α=0** | **α** | **(95% CI)** | ***p*-value for LR test α=0** |
|  | | Over-dispersion parameter | | 1.83 | (1.13, 2.97) | <0.001 | 1.58 | (0.94, 2.63) | <0.001 |
|  |  | |  |  |  |  |  |  |  |
|  | |  | | **Unadjusted** | | | **Adjusted for study, sex, age** | | |
|  | | **Consultations** | | **IRR** | **(95% CI)** | ***p*-value** | **IRR** | **(95% CI)** | ***p*-value** |
| Group |  | | Intervention vs Control | 1.01 | (0.92, 1.10) | 0.84 | 1.01 | (0.93, 1.10) | 0.82 |
| Study |  | | PACE-UP vs PACE-Lift |  |  |  | 0.85 | (0.77, 0.95) | 0.004 |
| Sex |  | | Male vs Female |  |  |  | 0.87 | (0.80, 0.95) | 0.001 |
| Age |  | | per year |  |  |  | 1.02 | (1.02, 1.03 | <0.001 |
|  |  | |  | **α** | **(95% CI)** | ***p*-value for LR test α=0** | **α** | **(95% CI)** | ***p*-value for LR test α=0** |
|  | | Over-dispersion parameter | | 0.54 | (0.50, 0.59) | <0.001 | 0.51 | (0.47, 0.56) | <0.001 |
|  | |  | |  |  |  |  |  |  |

**S1_Table: Coefficients for Cox regression and negative binomial models**
