# Supplementary material for: Effect of pedometer-based walking interventions on long-term health outcomes: Prospective 4-year follow-up of two randomised controlled trials using routine primary care data
Source: PLoS Med. 2019 Jun 25;16(6):e1002836. doi: 10.1371/journal.pmed.1002836 (PMC6592516; doi:10.1371/journal.pmed.1002836)
Supplement: S2 Table — (DOCX) [file pmed.1002836.s008.docx]

**Table S2: Details of physical activity intervention studies with robust health outcomes**

**A: Physical activity trials with cardiovascular outcomes**

| **Study** | **Summary of trial design and intervention** | **Participant details** | **Effect of intervention on physical activity levels and on cardiovascular events at long-term follow-up** | **Additional comments** |
| --- | --- | --- | --- | --- |
| **Arija et al 2017[1]** Effectiveness of a PA program on cardiovascular disease risk in adult primary care users: the Pas-a-Pas community intervention trial. | Multicentred RCT group-based intervention lasting 9 months (2x/week walking, 60 mins each + 1/month social gathering) compared to usual care control group. Collected risk factors at baseline & after intervention and cardiovascular events 2 years later. | Adult Spanish primary care attenders mean age 65-67 years.  N=419 patients (114 control, 305 intervention) from 4 primary care centres. 87% completed programme. | Intervention reduced blood pressure & cholesterol, increased self-report PA levels at 9 months (intervention group increased by 1132 METs/min/week relative to control, p=0.03). 2 years post intervention: 73% of intervention & 27% of control groups reported adhering to PA guidelines (p=0.01); and 5/260 (2.5%) of the intervention group had cardiovascular events (2 AMI & 3 CVA) compared to 10/104 (10.4%) of control group (8 AMI & 2 CVA) 2.5% (relative risk 0.15 (95% CI 0.04-0.51). Did not include cardiovascular deaths. | Cardiovascular events were assessed by interrogating the primary care computerised records and hospital database for all those followed up, 364/419 (87%), not for all those randomised. Not clear if this was conducted blind to treatment group. |
| **Newman et al 2016[2]**  Cardiovascular events in a PA intervention compared with a successful aging intervention: the LIFE study randomized trial. | Multicentred RCT comparing structured moderate intensity PA, 2 centre-based visits/week & home based activity 3-4x/week for 2.6 years compared to successful aging group, receiving weekly health education for 6 months then monthly for 2.6 years. Assessed cardiovascular disease (CVD) events (fatal/ non fatal myocardial infarction (MI), angina, stroke, transient ischaemic attack (TIA) and peripheral vascular disease. Limited outcome of MI stroke & cardiovascular death also studied. | 8 field centres across US, 1635 sedentary men and women aged 70-89 with some functional impairment (a Short Physical Performance Battery (SPPB) score of 9 or less). High baseline prevalence of cardiovascular disease 30%, hypertension 69% and diabetes 26% at baseline. | Follow-up 2.6 years. Intervention significantly increased PA levels by 40 minutes per week (95% CI 29-52) of moderate PA in intervention relative to successful aging control group, but cut-off for moderate PA was >760 counts/min) and significantly reduced motor disability (Hazard ratio 0.82 (0.69-0.98). However, there was no reduction in cardiovascular events in PA group cf successful aging. New CVD events in 121/818 (14.8%) of PA and 113/817 (13.8%) of successful aging participants. HR 1.1 (95% CI 0.85-1.42). For MI/stroke/CV death rates were 4.6% PA 4.5% successful aging, HR 1.05 (95% CI 0.67-1.66). | Participants were questioned at 6m contacts re hospital admissions since last contact, hospital records obtained to verify diagnoses, assigned blind to group. Deaths tracked through surveillance, silent MI assessed by ECG at baseline, 18m & 36m. Possible reasons given for lack of cardiovascular effect: dose of activity suboptimal (PA change lower than our trial, as lower moderate PA level cut-offs); high burden of CVD- too late to benefit and may have precipitated more events; more frequent contact by PA group could have reported more events. |
| **Gong et al 2015[3]** Efficacy of a community-based PA program KM2H^2^ for stroke & heart attack prevention among senior hypertensive patients: a cluster phase II RCT. | Cluster RCT, intervention was 6 sessions over 3 months (2 lectures, 2 telephone counselling sessions & 2 group meetings for understanding high blood pressure & increasing PA levels) plus 2 booster sessions from 3-6 months. Control group received standard clinical care. Outcomes assessed at 3 & 6 months: blood pressure (measured); PA (self-report); heart attack & stroke (reported by patients at follow-up and verified in clinical records). | N=450 participants aged ≥55 with hypertension from 12 community health centres in China (intervention 6 centres, n=232, control 6 centres, n=218). 381 (85%) followed up at 3m and 355 (79%) at 6m. | Outcomes assessed 6 months from baseline. Intervention group showed higher self-reported PA levels (type of activity, frequency and duration) (Cohen’s d=0.45 95% CI 0.04 to 0.85) and lower blood pressure (-3.7mm in SBP and -2.9mm in DBP). Intervention group versus control also showed reduced incidence of heart attack (3.6% vs 7.0% (p<0.05) & stroke (5.1% vs 9.9% (p<0.05). Cardiovascular deaths were not included. | Very high levels of events (but older hypertensive patients). Events were asked about at assessment periods and then medical records were checked for those who reported events (could be reporting bias). Did not routinely check all records for events. Note that controls had higher smoking rates than intervention 24% vs 14% but models adjusted for smoking. |

**B: Physical activity trials with new onset type 2 diabetes as outcome**

| **Study** | **Summary of trial design and intervention** | **Participant details** | **Effect of intervention on physical activity levels and on type 2 diabetes onset at long-term follow-up** | **Additional comments** |
| --- | --- | --- | --- | --- |
| **Tuomilehto et al 2001[4]**  Prevention of type 2 diabetes mellitus by changes in lifestyle among subjects with impaired glucose tolerance.  (Finnish Diabetes Prevention Study) | Patients with impaired glucose tolerance in Finland randomly assigned to either intervention (lifestyle: PA and diet counselling) or control. Intervention aimed to reduce weight, total intake of fat and intake of saturated fat and to increase intake of fibre and PA. Mean follow-up was 3.2 years. Assessed the incidence of type 2 diabetes. | 522 adults aged 40 to 65y with impaired glucose tolerance | Mean weight loss between baseline and 1 year was 4.2 kg (+/- s.d. 5.1kg) in intervention group and 0.8kg (+/- s.d. 3.7kg) in control group (P<0.001), net loss by end of year 2 was 3.5 (+/-s.d. 5.5kg) in intervention and 0.8 (+/- 4.4kg) in control group by end of year 2 (p<0.001). PA was assessed by self-report, how many achieved >4 hrs/week of exercise, at end of year 1, intervention group 86% versus control group 71% (p=0.001). Risk of diabetes type 2 was reduced by 58% in the intervention group (p<0.001). | Lifestyle intervention was not purely a PA one but also included detailed tailored dietary advice. Did not use routine records for outcomes, assessed by regular trial measures including blood tests. |
| **Davies et al 2016[5]**  A community based primary prevention programme for type 2 diabetes integrating identification and lifestyle intervention for prevention: the Lets prevent diabetes cluster RCT | In patients with prediabetes  44 General Practices randomised to receive either a structured educational programme or standard care. Primary outcome was progression to Type 2 DM during 3y. | 880 participants aged 40-75y screened and found to be at risk of pre-diabetes and diabetes | They measured PA by sealed pedometer and showed a significant increase in step-count 498 steps/day (95% CI 162 to 834) across all time points. (Increase at 3 years was 469 steps/day (95% CI 29 to 909). They also showed significant improvements in HbA1c, LDL cholesterol and sedentary time.  They showed a non-significant 26% reduced risk of developing T2DM in the intervention arm compared to standard-care HR 0.74 (95% CI 0.48 to 1.14) (p=0.18). The effect was greater (35% reduction) in the per-protocol analysis although still non-significant (p= 0.07). | Lifestyle intervention was not purely PA, but aimed at changes in diet to achieve weight loss, reduced saturated fat and increased fibre as well as increased PA.  Did not use routine data to collect outcomes. |

**C: Physical activity trials with fractures or falls as outcomes**

| **Study** | **Summary of trial design and intervention** | **Participant details** | **Effect of intervention on physical activity levels and on falls and fractures at long-term follow-up** | **Additional comments** |
| --- | --- | --- | --- | --- |
| **Zhao et al 2017[6]**  Exercise interventions and prevention of fall-related fractures in older people: a meta-analysis of randomised controlled trials. | RCTs that conducted exercise interventions and reported fall related fracture data in older people were included. | 15 studies including 3136 participants aged between 54 and 85 were included. | Exercise has a beneficial effect on the reduction of fall-related fractures with pooled estimate of RR 0.604 (95% CI 0.453 to 0.840) P=0.003, I^2^=0%. The rate of falls was also significantly reduced by exercise interventions RaR=0.856 (95% CI0.778 to 0.941) p=0.001, I^2^=45%. | Out of the 15 trials included, only a single trial was walking only. Several trials included walking alongside other types of intervention for strength and balance and resistance training and several trials did not include a walking component at all in their intervention. |
| **Okubo et al 2016[7]** Walking can be more effective than balance training in fall prevention among community-dwelling older adults. | 3 month supervised and 13 month unsupervised falls prevention programme. Randomly allocated into walking (brisk walking) group or balance (balance and strength training) group. Falls and trips that occurred were monitored with a monthly fall caldendar. | 90 adults aged 65-79 years. | The walking group significantly reduced their falls risk rate ratio 0.38 (95% CI 0.19 to 0.77) compared to the balance group. (Where fall was defined as “unintentionally coming to rest on the ground, floor or other lower level”. In contrast the number of trips (stumbling over an object without landing on any part of the body) significantly increased with walking rate ratio 1.5 (95% CI 1.12-2.0). | All data on falls and trips were self-reported. |
| **Iliffe et al 2015[8]** Promoting physical activity in older people in general practice: ProAct65+ cluster randomised controlled trial. | Randomised by practice into 3 arms: class-based Falls Management Exercise Programme (FAME), home-based Otago exercise programme (OEP) or usual care. Primary outcome was the proportion reaching recommended PA target 12 months post intervention. Secondary outcomes included self-reported falls. | 1256 people aged 65 years and over from 43 practices in London, Nottingham and Derby. | The FAME programme increased self-reported PA at 12 months post intervention (49% self-reporting achieving PA guidelines compared with 38% for usual care (p=0.02) and this was maintained at 24 months. FAME also significantly reduced self-reported falls at 12 months compared to usual care rate ratio 0.74 (95% CI 0.55-0.99). (OEP had no significant effect on self-reported PA or falls at 12 months). | No data from primary care records. Note this was an intervention specifically aimed at improving balance and falls rather than just increasing PA levels. |
| **Lawton et al 2008[9]** Exercise on prescription for women aged 40-74 recruited through primary care: two year RCT. | 2 year RCT of exercise prescription for women aged 40-74y in primary care. The prescription was provided by a GP or practice nurse and included encouraging moderate intensity physical activity such as brisk walking 30 minutes five days weekly. | 1089 women aged 40-74y | Intervention increased proportion self-reporting achieving PA guidelines of 150 minutes of MVPA weekly from 10% to 39% in the intervention group and from 11% to 33% in the control group (p<0.001). At 24 months: Increased falls in intervention group (179 events in intervention group vs 143 events in control group p<0.001). Increased injuries in intervention group (92 events in intervention group vs 66 events in control group p<0.03). | The data on physical activity and on falls and injuries were all based on recall. The authors note, “Adverse events of falls and injuries were self-reported so were open to recall bias.” |

**D: Physical activity trials with depression as outcome**

| **Study details** | **Summary of trial design and intervention** | **Participant details** | **Effect of intervention on physical activity levels and on depression / depressive symptoms at long-term follow-up** | **Additional comments** |
| --- | --- | --- | --- | --- |
| **Conn 2010[10]**  Depressive symptom outcomes of physical activity interventions: meta-analysis findings | Performed a meta-analysis looking at depressive symptom outcomes among health adults. Analyses included random effects standardised means. | Included 38 supervised PA studies and 22 unsupervised PA studies. | This meta-analysis yielded a standardised mean effect size of 0.372 (95% CI 0.242 to 0.503) amongst 38 supervised PA interventions and 0.522 (95% CI 0.277 to 0.767 among 22 unsupervised PA studies. | Preliminary moderator analyses suggested that supervised interventions including flexibility / resistance and low intensity exercise may be more effective and unsupervised interventions may be more effective when they recommend centre based PA. |
| **Pavey et al 2011[11]**  Effect of exercise referral schemes in primary care on physical activity and improving health outcomes: systematic review and meta-analysis. | Performed a systematic review and meta-analysis, included 8 RCTs. | 6 trials compared exercise referral schemes with usual care, 2 compared them with an alternative PA intervention. One compared exercise referral scheme with a self-determination theory intervention. | Compared with usual care, exercise referral schemes showed an increased number of participants who self-reported achieving 90-150 minutes of MVPA weekly RR 1.16 (95% CI 1.03 to 1.30) and a reduced level of depression (pooled standard mean difference -0.82 (95% CI -1.28 to -0.35). No difference was found between exercise referral schemes and the other two comparator groups. | They concluded that considerable uncertainty still remains about the effectiveness of exercise referral schemes for increasing PA, fitness or health indicators. |

**References**

1. Arija, V., et al., *Effectiveness of a physical activity program on cardiovascular disease risk in adult primary health-care users: the "Pas-a-Pas" community intervention trial.* BMC Public Health, 2017. **17**(1): p. 576.

2. Newman, A.B., et al., *Cardiovascular Events in a Physical Activity Intervention Compared With a Successful Aging Intervention: The LIFE Study Randomized Trial.* JAMA Cardiol, 2016. **1**(5): p. 568-74.

3. Gong, J., X. Chen, and S. Li, *Efficacy of a Community-Based Physical Activity Program KM2H2 for Stroke and Heart Attack Prevention among Senior Hypertensive Patients: A Cluster Randomized Controlled Phase-II Trial.* PLoS One, 2015. **10**(10): p. e0139442.

4. Tuomilehto, J., et al., *Prevention of type 2 diabetes mellitus by changes in lifestyle among subjects with impaired glucose tolerance.* N Engl J Med, 2001. **344**(18): p. 1343-50.

5. Davies, M.J., et al., *A community based primary prevention programme for type 2 diabetes integrating identification and lifestyle intervention for prevention: the Let's Prevent Diabetes cluster randomised controlled trial.* Prev Med, 2016. **84**: p. 48-56.

6. Zhao, R., F. Feng, and X. Wang, *Exercise interventions and prevention of fall-related fractures in older people: a meta-analysis of randomized controlled trials.* Int J Epidemiol, 2017. **46**(1): p. 149-161.

7. Okubo, Y., et al., *Walking can be more effective than balance training in fall prevention among community-dwelling older adults.* Geriatr Gerontol Int, 2016. **16**(1): p. 118-25.

8. Iliffe, S., et al., *Promoting physical activity in older people in general practice: ProAct65+ cluster randomised controlled trial.* Br J Gen Pract, 2015. **65**(640): p. e731-8.

9. Lawton, B.A., et al., *Exercise on prescription for women aged 40-74 recruited through primary care: two year randomised controlled trial.* BMJ, 2008. **337**: p. a2509.

10. Conn, V.S., *Depressive symptom outcomes of physical activity interventions: meta-analysis findings.* Ann Behav Med, 2010. **39**(2): p. 128-38.

11. Pavey, T.G., et al., *Effect of exercise referral schemes in primary care on physical activity and improving health outcomes: systematic review and meta-analysis.* BMJ, 2011. **343**: p. d6462.
